# Fewer Dimensions, More Structures for Improved Discrete Models of Dynamics of Free versus Antigen-Bound Antibody

**DOI:** 10.3390/biom12071011

**Published:** 2022-07-21

**Authors:** Kazi Lutful Kabir, Buyong Ma, Ruth Nussinov, Amarda Shehu

**Affiliations:** 1 Department of Computer Science, George Mason University, Fairfax, VA 22030, USA; amarda@gmu.edu; 2 Engineering Research Center of Cell & Therapeutic Antibody School of Pharmacy, Shanghai Jiaotong University, Shanghai 200240, China; mabuyong@sjtu.edu.cn; 3 Computational Structural Biology Section, Cancer Innovation Laboratory, Frederick National Laboratory for Cancer Research, National Cancer Institute, Frederick, MD 21702, USA; nussinor@mail.nih.gov

**Keywords:** antibody, antigen binding, structure, molecular dynamics, Markov State Model

## Abstract

Over the past decade, Markov State Models (MSM) have emerged as powerful methodologies to build discrete models of dynamics over structures obtained from Molecular Dynamics trajectories. The identification of macrostates for the MSM is a central decision that impacts the quality of the MSM but depends on both the selected representation of a structure and the clustering algorithm utilized over the featurized structures. Motivated by a large molecular system in its free and bound state, this paper investigates two directions of research, further reducing the representation dimensionality in a non-parametric, data-driven manner and including more structures in the computation. Rigorous evaluation of the quality of obtained MSMs via various statistical tests in a comparative setting firmly shows that fewer dimensions and more structures result in a better MSM. Many interesting findings emerge from the best MSM, advancing our understanding of the relationship between antibody dynamics and antibody–antigen recognition.

## 1. Introduction

With AlphaFold2 advancing protein structure determination to computational models that now reach the accuracy of experimentally-resolved models [1,2], several next holy grails of computational biology are ordering themselves [3]. Primary among them is the much-needed generalization from the single- to the multi-structure view of protein molecules; obtaining a detailed view of the structural dynamics of unbound and bounded molecular systems remains a fundamental challenge [4].

Over the past decade, Markov State Models (MSM) have emerged as powerful methodologies to build discrete models of dynamics over trajectories obtained in silico via Molecular Dynamics (MD) simulation [5,6]. Most applications of MSMs in molecular biology literature have operated over the structure space probed via MD trajectories and have ignored energy information [6,7,8,9,10,11], utilizing clusters over feature-based representations of structures as macrostates.

Connections with the energy landscape that governs the structural dynamics of a molecule have largely been ignored, even though the concept of a basin in the energy landscape promises to connect more directly to that of a macrostate. The reasons vary and include the long-standing lack of trust in the accuracy of energy functions and, perhaps, more importantly, the open questions between connecting the definition of a basin to practical implementations over a set of structure–energy samples.

Work in [12,13] leverages a nearest-neighbor graph [14] to organize structure–energy samples into basins [15] and demonstrates for the first time their employment as macrostates of an MSM of the MD-probed dynamics of a small peptide, Met-Enkephaline. Work in [16] extends this treatment to elucidate changes in dynamics due to binding and understand the impact of antigen binding on the dynamics of Immunoglobulin G (IgG) but is challenged by the large size of the molecular system under consideration, the free and the antigen-bound antibody. The work considers several approaches to simplify the representation of a free (tertiary) and complexated (quaternary) structure and ultimately shows that a simple representation that skips every few consecutive central (alpha) carbon atoms is more effective than representations obtained via dimensionality reduction methods popular for MSM construction, such as Principal Component Analysis (PCA) or Time-structure Independent Component Analysis (TICA). However, the dimensionality of the representation affects both the quality of obtained structure clusters (where no energy is considered) and obtained basins (when energy is considered) and ultimately results in MSMs that, while informative of the free versus bound antibody dynamics, but do not stand to the scrutiny of all statistical tests of model quality [16].

Motivated by this body of work and practical challenges, we posit here the following: (a) further reducing the representation dimensionality in a non-parametric, data-driven manner may improve the quality of an MSM by providing better state space discretization; (b) including more structures in the computation is then possible and promises a higher quality MSM even without the access to energetics.

This paper investigates these two research directions. It does so by bringing back a shape-based representation of a molecular tertiary structure, the Ultrafast Shape Recognition (USR) coordinates [17]. These coordinates were originally proposed for molecular shape comparison and shown to permit fast screening of billions of small molecule compounds without the need to align molecular structures before testing for similarity. USRs were shown to be highly accurate at describing and comparing molecular shapes. Work in [18] further showed that USR coordinates were highly useful for low-dimensional representations of protein tertiary structures, and a body of work utilized them to build fast, search space-aware algorithms for exploring and reconstructing the structure space of a protein molecule [19,20], as well as computing motion paths connecting two given structures of a protein molecule [21].

The context that motivates the computational study in this paper is that of aiming to understand the impact of antigen binding on the dynamics of Immunoglobulin G (IgG), which binds to cognate antigens. Section 2 summarizes current literature and understanding on IgGs and their binding of antigens. This section also summarizes MSM methodologically for the interested reader. Methodological details are related in Section 3. Section 4 relates the experimental evaluation. In essence, the evaluation compares the quality of three MSMs, one that is presented in [16], and the two obtained by each of the approaches outlined above. Rigorous statistical testing is conducted to evaluate the quality of an MSM. Several interesting observations emerge. In particular, the evaluation firmly shows the superiority of the models obtained when USR coordinates are used to represent structures. In particular, the MSM obtained when more structures are included shows the power of the data over energetics. It is worth noting that in each case two separate analyses are carried out, one on the free antibody and the other on the antigen-bound antibody. Comparison of these two systems reveals interesting insight on how the antigen binding affects the antibody dynamics. The paper concludes with such observations in Section 5.

## 2. Background

### 2.1. Related Work on MSMs of Structural Dynamics

Since Zwanzig’s first report on short memory approximations in 1983 [22], MSMs have evolved from art to science [23]. Their theoretical underpinnings, rigorous statistical analysis, and availability as easy-to-use and modify packages are the primary reasons for their increase in popularity. MSMBuilder [24] and EMMA [25,26] are two of the most popular ones. PyEMMA provides a Python implementation of EMMA. Both MSMBuilder and EMMA are widely employed by researchers to compute the multi-state folding kinetics of small proteins and peptides [11], the kinetics of protein-ligand binding for drug efficacy [9], the kinetics of protein-protein associations [7,8], and more [6,10].

As in our previous work [12,16], we make use of PyEMMA [26], as it is easy to use, contains many analysis tools, and provides developers with control over various steps of the MSM construction pipeline. In particular, PyEMMA allows us control the feature representation of structures and the computation of macrostates. These are distinct stages in the MSM construction pipeline.

The identification of macrostates for the MSM is a critical component. According to theory, structures capable of rapid inter-conversion should be aggregated into the same macrostate; structures with slow inter-conversion rate should be separated into different macrostates [27]. In practice, MSM construction packages like PyEMMA cluster conformations to group them into macrostates. They make use of the assumption that a molecule converts/switches between geometrically-similar structures more rapidly than between less similar ones.

Work in [12,16] operationalizes the realization that conformations proximal in structure space can be separated by an energy barrier that slows the inter-conversion rate. Thus, the concept of basin is employed instead for that of a macrostate, and the identification of basins utilizes both structures and their energies.

### 2.2. Molecular System of Interest

The particular systems that motivate the work presented in this paper are the IgGs, which are central in providing us with immunity to pathogens and tumors. As such, they represent common template for antibody drugs [28]. We now understand that the specificity and affinity of antibody–antigen recognition is primarily determined by the complementarily-determining regions (CDRs), which are variable domains. The recognition process relies on conformation transitions that are mediated by the antibody’s inherent flexibility [29,30,31,32,33].

Several studies suggest that allosteric effects during antibody–antigen recognition [34] with both the variable and constant domains play an important role [35,36,37,38,39,40]. In particular, modifications of the Fc domain influence the Fv domain antigen recognition. Upon antigen binding, the C domains may affect the V region paratope conformation, as indicated by circular dichroism [41], X-ray crystallography [42], and NMR [43]. Modification of the constant domain influences both the antibody–antigen binding affinity [44,45,46] and specificity [47,48]. Work in [49] shows that IgA Fc mutations reduce Her2 binding, and work in [32] shows that antigen binding allosterically promotes Fc receptor recognition.

#### 2.2.1. Free Antibody and Antigen-Bound Antibody Structure Data

The MD simulations from which we extract structures for analysis in this paper are reported in detail in [32]. Conclusions obtained in [32] on antibody structure distributions were verified by independent experiments; as reported in [50], the MD simulation results in [32] reproduce experimentally-observed antibody structure distributions.

MD Simulation Protocol: We briefly review the MD protocol in [32]. The structure of the free antibody molecule, which can be downloaded from the Protein Data Bank (PDB) from PDB entry 1IGT, contains 1322 amino acids for a total of 20,544 atoms. The antigen–antibody complex contains 21,092 atoms (1356 amino acids). Work in [32] adjusts three sets of torsion angles (231C-232N-232CA-232C, 232N-232CA-232C-233N, and 232CA-232C-233N-233CA) with increments of 60° to obtain initial antibody random structures. The Fc domain is kept fixed during this process, whereas the Fab domains are allowed to move freely. A total of 216 structures are obtained in this manner. Removing those with a closed Fab domain or with collisions in the Fc domain leaves us with 12 structures as starting points for MD simulations.

Work in [32] carried out 12 independent MD simulations of the antibody with 12 different initial structures. Each MD trajectory is 40–160 ns long, with a time step of 2 ps between two consecutive frames/structures.

The systems were solvated by TIP3P water molecules, and sodium and chlorides were added to neutralize the system and to achieve a total concentration of 150 mM. The overall sizes of simulated systems contain around 567,640 atoms (including water atoms as solvent). The systems were energy minimized for 5000 conjugate gradient steps, where the protein was fixed and water molecules and counterions could move, followed by additional 5000 conjugate gradient steps, where all atoms move. In the equilibrium stage, each system was gradually relaxed by a series of dynamic cycles, in which the harmonic restraints on proteins were gradually removed to optimize the protein–water interactions. In the production stage, all simulations were performed using the NPT ensemble at 310 K. All MD simulations were performed using the NAMD [51] software with CHARMM36 [52] force field. MD trajectories were saved by every 2 ps for analysis. The structures obtained from simulation reproduced experimentally-observed structure distribution [50]. The potential energy of the system was calculated using the generalized Born method with molecular volume (GBMV) after the steps of energy minimization to relax the local geometries caused by the thermal fluctuations that occurred in the MD simulations. In the GBMV calculation, the dielectric constant of water is set to 80, and no distance cutoff is used. Further details regarding the process of structure generation can be found in [32]. Internal energies were only recorded for 5000 structures (every 32nd frame).

Structures are extracted with the mdconvert command-line script from the MDTraj Python library over a given MD trajectory file in the .dcd format. We extract frames every 4 ps, resulting in a total of 160,000 structures for the free antibody and the antibody–antigen complex, respectively.

#### 2.2.2. Prior MSM-Based Investigation

The large sizes of the free and bound antibody prompt work in [16] to consider several representation reduction strategies and to focus on only the 5000 structure–energy samples for each system (free and bound). Thorough analysis shows that simplifying a structure to a high-dimensional point $(CA_{1}.x,CA_{1}.y,$$CA_{1}.z,CA_{5}.x,CA_{5}.y,CA_{5}.z,CA_{10}.x,CA_{10}.y$, $CA_{10}.z,\ldots )$ (effectively skipping every four consecutive *CA* atoms) yields the best MSM model; however, all MSM models have various shortcomings in relation to diverse rigorous statistical tests.

## 3. Methods

We first relate the USR-based representation that we propose as fast yet accurate in this paper. We then place it in the context of the process that harnesses MD trajectories into an MSM model of dynamics. Finally, we describe the various statistical tests that evaluate the quality of an MSM model and set the stage for the evaluation related in Section 4.

### 3.1. Shape-Based Coordinates/Features

In MSM terminology, the coordinates employed to represent a tertiary or quaternary structure are more generally referred to as *features*, and the process via which such features are obtained is referred to as *featurization*. Whether in the free antibody or the antigen-bound antibody setting, we represent a structure as a 12-dimensional point, utilizing the following process to compute 12 coordinates from a given structure. First, given a structure in three dimensions, four points of reference are identified over it:

1. The molecular centroid (ctd);
2. The closest atom to ctd (cst);
3. The furthest atom from ctd (fct);
4. The furthest atom to fct (ftf).

The Euclidean distances of all the atoms in a structure to each of the four reference points yield four separate distance distributions. Each distribution is summarized with its first three moments (mean, variance, and skewness), yielding over the four distributions the 12 coordinates/features representing a structure and thereby capturing the geometry and shape of the structure.

Note that this representation presents a significant reduction from several thousand Cartesian coordinates to 12 (USR) coordinates. Given the known curse of dimensionality for clustering algorithms [53], such savings promise to translate to better-quality clusters for large molecular systems.

### 3.2. Integrating MD Trajectories in an MSM

**Figure 1:**
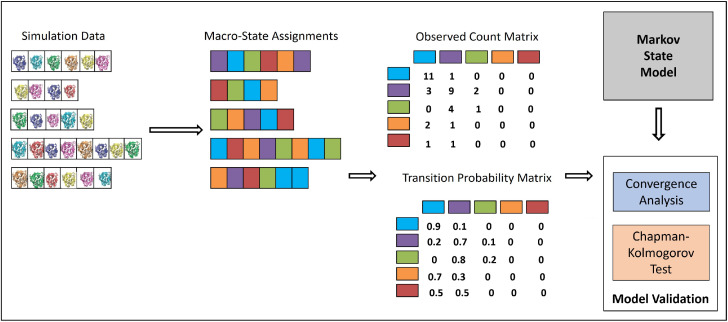
The computational pipeline that “integrates” the information available in the MD trajectories into an MSM of the system’s dynamics. The different color codes are representing the different macro-states/groups; we also color-code the structures to illustrate their assignments to specific macro-states.

Figure 1 summarizes the process that integrates MD trajectories into an MSM transition probability matrix where each entry contains the probability of transition between each pair of the macrostates; the rows/columns are the identified macrostates.

The first MSM-construction decision is how to featurize the structures, which is addressed via the USR coordinates as described above. We recall that we compare here to an alternative featurization (shown as most effective in [16] that records the Cartesian coordinates of the CA atoms and skips every four consecutive CA atoms. From now on, let us make the distinction of conformation versus structure, employing the term conformation to a structure that has undergone the featurization process.

The second decision is how to group conformations into clusters/macrostates. In [16], the conformations are organized in a nearest-neighbor to identify basins. Each vertex is a conformation–energy pair. Vertices representing local (focal) minima are identified first: a vertex *u* is a local minimum if ∀v∈N(u) and ∀v∈V,e(u)≤e(v), where *e* denotes energy, and N(u) denotes the neighborhood of *u*. A basin is tied to a unique local minimum that is its deepest point. Vertices “attracted” to the same local minimum are assigned to the same basin represented by that local minimum. Each vertex *u* is associated with a negative gradient estimated by selecting the edge (u,v) maximizing the ratio [e(u)−e(v)]/d(u,v) (*d* is the distance between two vertices, measured in conformation space). Consider a vertex *u* that is not a local minimum. From each such vertex, there are many edges to follow. The one that maximizes the negative gradient calculated as shown above is selected to arrive at a next vertex. This process is repeated until we arrive at a local minimum. Vertices from which this process terminate at the same local minimum are then assigned to that minimum and the basin associated with it. Further details can be found in [54]. We note that basin finding over conformation-energy samples has been employed by several works for molecular structure–function studies [12,55,56,57].

In this paper, we utilize this approach to identify basins over conformation–energy samples obtained from the MD simulations. We recall that our conformations are 12-dimensional points (of USR coordinates). Unlike work in [16], where the CA-based representation necessitates the computationally-costly process of alignment and then rmsd-computation; here, we utilize Euclidean distance to measure the distance between two USR-based conformations. In this setting, where we consider energies, we utilize 5000 conformations for which energies are available, as in [16]. In another setting, where we investigate the benefit of including many more structures, we utilize all 160,000 structures collected. Utilizing their 12-dimensional, USR-based representation, we then make use of the popular k-medoid algorithm to identify clusters. Finding the knee in the squared error profile over varying *k* allows us to identify an optimal number of clusters in this setting.

Once the basins or clusters are obtained to be employed as macrostates, the next step is to utilize the temporal information in the MD trajectories to compute state-to-state probabilities of transition. Briefly, let us assume that some conformation *i* has been assigned to a macrostate $S_{i}$, and some other conformation *j* has been assigned to a macrostate $S_{j}$. If *j* follows *i* in some MD trajectory, the number of counts keeping track of transitions from $S_{i}$ to $S_{j}$ is incremented; note that $S_{i}$ and $S_{j}$ can be the same macrostate. Once all counts are computed, they are normalized to obtain transition probabilities. The eigenvalues and corresponding eigenvectors of the transition matrix bear meaning; the eigenvector whose corresponding eigenvalue has the maximum value of 1 gives the equilibrium/stationary distribution. We note that, in this stationary distribution, a high population for a macrostate indicates thermodynamic stability.

#### Statistical Tests of MSM Quality

As in other MSM-based studies of dynamics, we make use of two main tests, the Convergence Analysis and the Chapman–Kolmogorov (CK) test, which evaluate whether an MSM is memory-less. In a memory-less MSM, the conditional probability distribution of future macrostates should only depend on the current macrostate and not on the prior macrostates.

##### Convergence Analysis

The convergence analysis tests whether the selected lag time guarantees that the assignment of conformations in macrostates obeys the Markov property. This selection is an important decision, as it may yield to a loss of temporal and spatial resolution and in turn impact the quality of the constructed MSM. If the assignment of conformations into mascrostates obeys the Markov property, this means that conformations within a state inter-convert to other conformations in the same state on timescales faster than the selected lag time but transition to conformations in other states on slower timescales. This property is verified via visualization of the generated implied timescale plot of the model relaxation timescale versus model lag time. One looks for an exponential decay to the system equilibrium; the implied timescales plot needs to exhibit convergence within a few steps [58].

##### CK Test

The Chapman–Kolmogorov (CK) test computes the probability of transition between meta-stable states for increasing lag times. Given an MSM estimated at lag time τ, a quantity of interest can then be predicted at lag time kτ, and the prediction can be compared to an independently-estimated model at kτ. CK test plots need to show that the estimated and the predicted model deviate negligibly from each-other. The CK test is important because the estimated MSM dynamics can fluctuate in a deterministic manner from the actual dynamics of a system of interest. Work in [59] shows that this deviation may be present even when accounting for and removing statistical error by sampling more structures [59]. Specifically, the propagation error on the discrete space over identified macro-states is calculated by checking whether the approximation, ${\left[\hat{T}\left(\tau \right)\right]}^{k}\approx \hat{T}\left(k\tau \right)$ holds within statistical uncertainty. Note that $\hat{T}\left(\tau \right)$ is the state-to-state transition matrix estimated at the selected lag time τ; $\hat{T}\left(k\tau \right)$ is the state-to-state transition matrix estimated at longer lag times kτ. The Chapman–Kolmogorov (CK) test generalizes this check.

## 4. Results

### 4.1. Evaluation Setup

We consider two separate systems: the free antibody and the antigen–antibody complex. For each system, we construct three separate MSMs based on the combination of featurization choice and clustering algorithm.

Setting-A: This setting utilizes the CA-based representation and basin finding over 5000 structure–energy samples; as reported in [32], energies are recorded only over 32nd frame, resulting in a total of 5000 energy-evaluated structures. We employ this model presented as best in [16] as a baseline model for each setting.

Setting-B: The second setting changes the CA-based representation to the USR-based representation of 12 coordinates per structure to represent those 5000 structure–energy samples.

Setting-C: The third model expands to 160,000 structures (for all but 5000 of which there is no information on energies) and employs the k-medoidsalgorithm.

Each model (corresponding to each setting) is evaluated via the convergence analysis and CK tests. The best MSM in each setting is related in greater detail, providing insights on the dynamics of the free and bound antibody and changes to dynamics upon antigen binding.

### 4.2. Comparison of Models of Free Antibody Dynamics

We now evaluate each of the three MSMs obtained on the free antibody data via the convergence analysis and CK tests to determine the best model capturing the free antibody dynamics.

#### 4.2.1. Free Antibody MSM Evaluation via the Convergence Analysis

**Figure 2:**
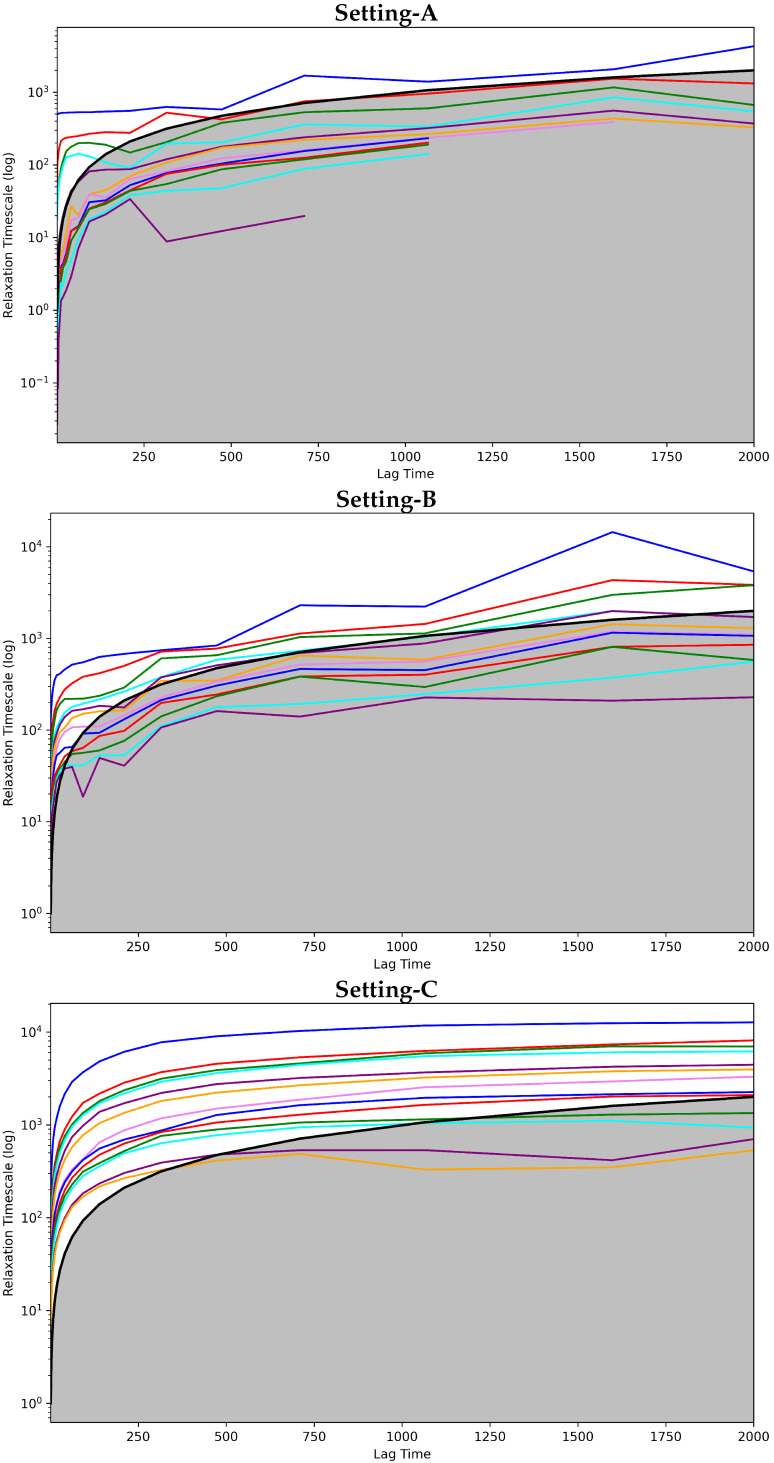
The Convergence Analysis test is carried out on each of the free-antibody MSMs obtained respectively from (**top** panel) Setting-A, (**middle** panel) Setting-B, and (**bottom** panel) Setting-C. Each drawn curve shows the relaxation timescale of an MSM composed of *s* states, as *s* varies from 1 to the total number of states identified. The cutoff region (above which any curve representing a good discretization of the state space should be) is shown in gray.

Figure 2 shows the implied timescales plots computed for each of the three MSMS. These plots show the model relaxation timescale versus the model lag time. The top panel of Figure 2 does so for Setting-A, the middle panel for Setting-B, and the bottom panel for Setting-C. In each panel, each drawn curve shows the relaxation timescale computed when considering an MSM composed of *s* states, with *s* varying from 1 to the total number of states identified.

Figure 2 clearly shows that Setting-C yields an MSM model that stands up best to the convergence analysis test. The other two settings fail to attain convergence even after 1500 steps. Rough oscillations in the curves are observed. In contrast, when the MSM is obtained when states identified via Setting-C, convergence is reached quite early. Figure 2 shows that the cutoff region above which any curve representing a good discretization of the state space should be is in gray. It is clear that only in Setting-C are the majority of the curves above this region. Taken altogether, this test clearly puts forth Setting-C as the best one.

#### 4.2.2. Free Antibody MSM Evaluation via the CK Test

**Figure 3:**
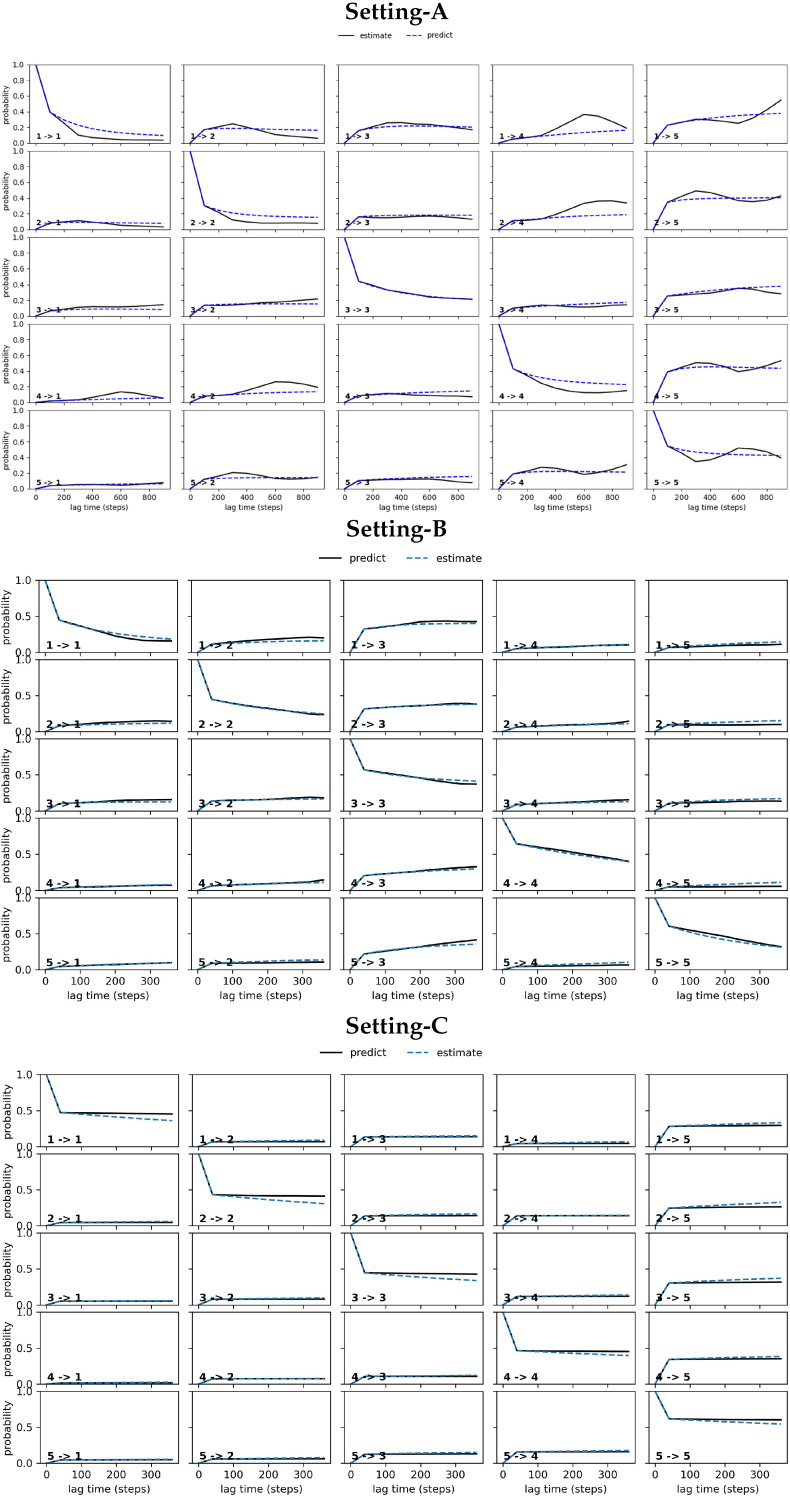
The CK test is carried out on each of the free antibody MSM models obtained, respectively, from (**top** panel) Setting-A, (**middle** panel) Setting-B, and (**bottom** panel) Setting-C. Deviations between the estimated and the predicted model are large in Setting-A, gradually reduce over Setting-B, and become negligible in Setting C.

Figure 3 relates the results obtained from conducting the CK test over the MSM models obtained respectively from Settings A–C. Specifically, Figure 3 visualizes the outcome for the five states identified in each setting that have the highest self-transition probabilities. The plots show deviations between the estimated and the predicted model in Settings A–B (top and middle panels). These are largest under Setting-A and gradually reduce over Setting-B and Setting-C; the deviations are negligible for Setting-C. In good agreement with the convergence analysis test related above, the CK test additionally confirms that the MSM model obtained from Setting-C is the best, as it stands up best to both of these statistical tests.

### 4.3. Comparison of Models of Antigen-Bound Antibody Dynamics

We now evaluate each of the three MSMs obtained on the antigen-bound antibody data via the convergence analysis and CK tests to determine the best model capturing the free antibody dynamics.

#### 4.3.1. Antigen-Bound Antibody MSM Evaluation via the Convergence Analysis

**Figure 4:**
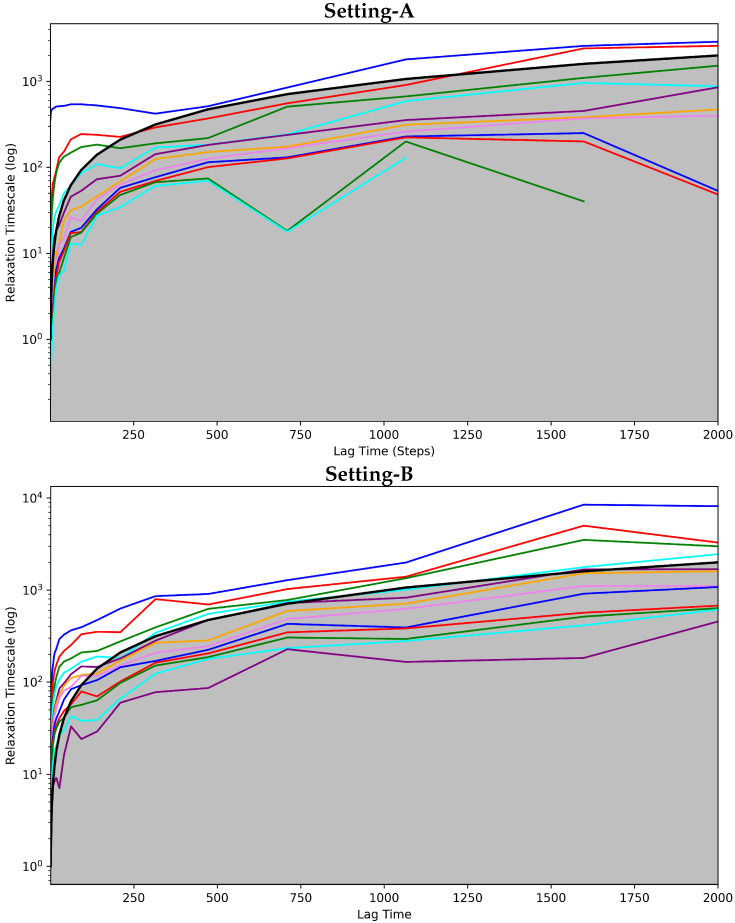

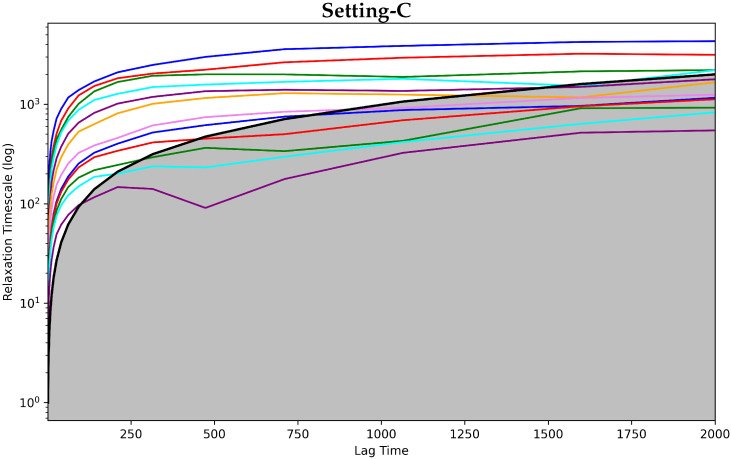
The Convergence Analysis is carried out on each of the antigen-bound antibody MSM models obtained, respectively, from (**top** panel) Setting-A, (**middle** panel) Setting-B, and **(bottom** panel) Setting-C. Each drawn curve shows the relaxation timescale of an MSM composed of *s* states, as *s* varies from 1 to the total number of states identified. The cutoff region (above which any curve representing a good discretization of the state space should be) is shown in gray.

Figure 4 shows the implied timescales plots computed for each of the three MSMS corresponding to Settings A–C. Figure 4 clearly shows that the models corresponding to Setting-A and Setting-B fail to attain convergence even after 1500 steps. Additionally, rough oscillations are observed in the curves. The majority of the curves are under the cutoff region that separates good from poor state space discretization. In contrast, Setting-C exhibits convergence early on. With very few exceptions, the curves have no rough oscillations, and the majority of them are over the cutoff region. Taken altogether, this test also puts forth Setting-C as the best one capturing the dynamics of the bound antibody.

#### 4.3.2. Antigen-Bound Antibody MSM Evaluation via the CK Test

**Figure 5:**
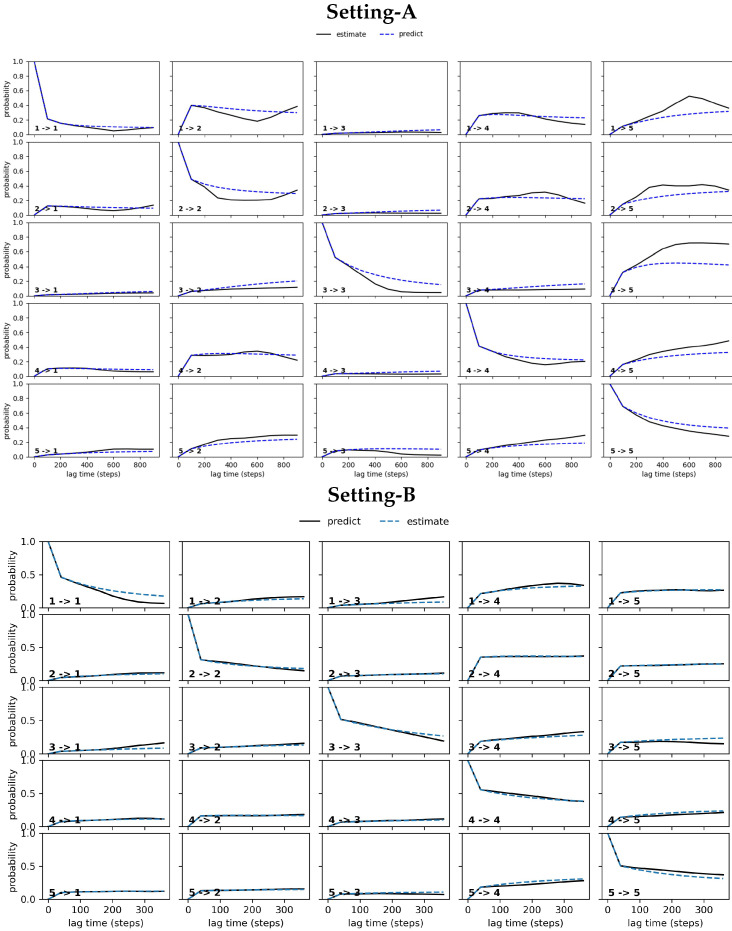

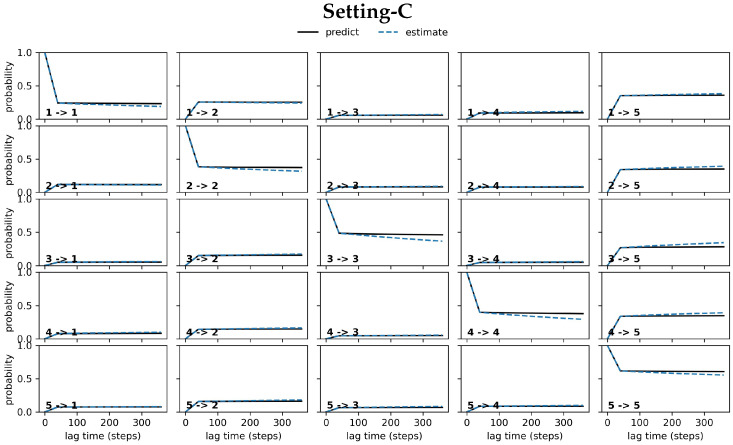
The CK test is carried out on each of the antigen-bound antibody MSM models obtained respectively from (**top** panel) Setting-A, (**middle** panel) Setting-B, and (**bottom** panel) Setting-C. Deviations between the estimated and the predicted model are large in Setting-A, gradually reduce over Setting-B, and become negligible in Setting C.

Figure 5 relates the results obtained from conducting the CK test over the MSM models obtained respectively from Settings A–C for the antigen-bound antibody. As for the antigen-antibody complex, Figure 5 visualizes the outcome for the five states identified in each setting that have the highest self-transition probabilities. The plots show deviations between the estimated and the predicted model in Settings A–B (top and middle panels). Figure 5 shows that these are largest under Setting-A and gradually reduce over Setting-B and Setting-C. Again, the deviations are negligible for Setting-C, as for the free antibody. In good agreement with the convergence analysis test related above, the CK test additionally confirms that the MSM model obtained for the antigen-bound antibody from Setting-C stands up best to both of these statistical tests.

#### 4.3.3. Run-Time Comparison

We relate a run-time comparison in Table 1, which shows the time it takes to construct an MSM under each setting (A–C) for each of the systems (free antibody versus antigen-bound). It can be clearly observed that Setting-C yields the largest run-time savings. Even though the number of structures processed goes from 5000 to 160,000 in Setting-C, the USR-based coordinates greatly reduce the time it takes to cluster structures and identify macrostates for the MSM.

### 4.4. Comparison of Free vs. Antigen-Bound Antibody Dynamics

According to the Convergence Analysis and CK tests, as well the run-time analysis, the best MSM obtained for both the free and antigen-bound antibody is that obtained from Setting-C. In the next set of analyses, we focus on the MSM model obtained from this setting.

#### 4.4.1. Macro-State Analysis: Comparison of Stationary State Distributions

**Figure 6:**
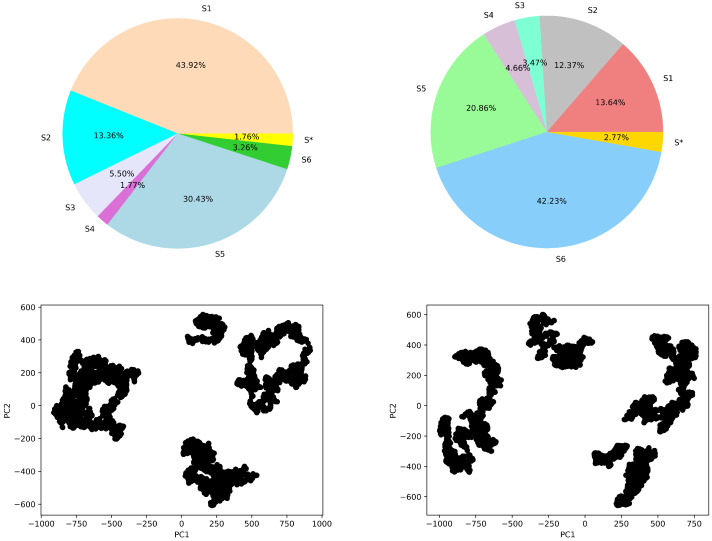
Top panel shows adjusted state populations for the (**left**) free-antibody and (**right**) antigen-bound antibody via the stationary distribution for the six top-populated macro-states, with the other states accumulated in S*. The bottom panel relates the embedding of the MD structures over the top two PCs.

First, we shed light on the state space discretization in a comparative setting. The procedure described in Section 3 identifies 14 clusters/states over the free-antibody data and 13 clusters/states over the antigen-bound antibody data. The top panel of Figure 6 relates stationary distribution via a pie chart limited to the six most populous states (labeled as S1–6 for states). In each pie chart, the population of the remaining macrostates is accumulated and labeled as S*. The bottom panel shows the 2D embedding of the sampled structure data (every 16th frame) in each case (for the free and antigen-bound antibody) on the top two Principal Components (PCs) revealed by PCA (the top two PCs capture around 60% of the variance—data not shown).

The juxtapositions in Figure 6 allow for drawing several observations. First, for the free antibody, four states emerge with an adjusted population at equilibrium over 5%, and they seem to correspond well to the four major clusters visible in the PC1-PC2 embedding related in the bottom left panel of Figure 6. For the antigen-bound antibody, the number of states with adjusted population at equilibrium above 5% is also 4, and this qualitatively agrees with the four major clusters visible in the PC1-PC2 embedding related in the bottom right panel of Figure 6. Overall, the number of states with high self-transition probabilities is higher for the free antibody than the antigen-bound system. This suggests that the binding of the antigen makes the states more dynamic.

#### 4.4.2. State-to-State Dynamics: Comparison of State Transitions

**Figure 7:**
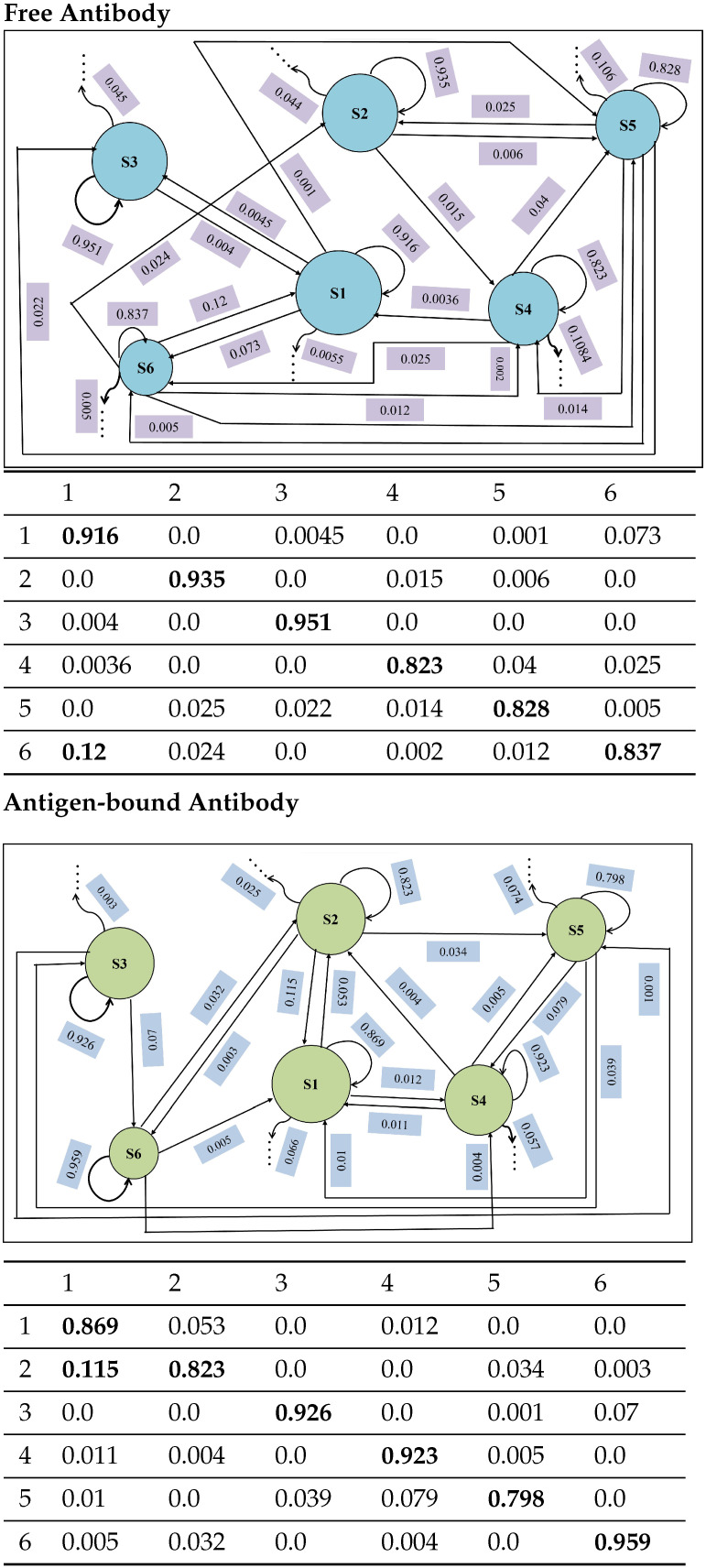
In each case, macro-states are drawn as disks, with radii proportional to size (number of conformations). Transitions between them are drawn as arrows, and transition probabilities are shown. The visual summary is restricted to the six top-populated states. Trailing arrows indicate transitions to other states. Transition probabilities for the six top-populated states (Values at or above 0.1 are highlighted in bold indicating considerable transitions).

Figure 7 juxtaposes the state-to-state transition probabilities for the free versus the antigen-bound antibody. In both cases, the self-transition probabilities are above 0.8 (except for S5 of the antigen-bound antibody with 0.798). For the free antibody, while the cumulative out-of-state transition probabilities for three states (S1, S2, S3) are below 0.09; state S6 transitions to state S1 with a considerable probability of 0.12. For the bound antibody, while the cumulative out-of-state transition probabilities for three states (S3, S4, S6) are below 0.08, state S2 transitions to state S1 with a considerable probability of 0.115. For the bound antibody, several of the self-transition probabilities are lower than for the free antibody. Taken altogether, the comparison of adjusted state populations and transition probabilities suggests that antigen binding makes the antigen–antibody complex more dynamic by introducing more inter-state transitions among the various states.

## 5. Conclusions

While the application setting that motivates the computational study in this paper is elucidating the impact of antigen binding on the dynamics of Immunoglobulin G (IgG), our study focuses on practical design choices that hamper the quality of MSMs integrating MD trajectories. Whether focusing on a free antigen or an antigen-bound one, the number of atoms in a molecular system can approach several hundred thousand. Even reducing to the central carbon atoms does not significantly reduce the number of atoms or dimensions. For the particular systems in this study, this number exceeds 20,000 atoms (for free antibody) and 21,000 atoms (for the antigen-bound antibody), respectively.

Such a high dimensionality of the structure space poses real challenges for the clustering algorithms on which MSM construction packages rely for state space discretization. In turn, the quality of MSMs suffers, as diagnosed by various statistical tests. Even employing dimensionality reduction techniques, such as PCA and TICA, does not promise a remedy [16]. In this paper, we show that reducing the representation dimensionality in a non-parametric, data-driven manner improves MSM quality. In particular, employing shape-based coordinates to featurize three-dimensional structures allows for including more structures in the computation, yielding a higher-quality MSM even in the absence of energetics. Furthermore, the Supplementary Figures S1–S12 demonstrate how diverse the structures are with respect to the initial/starting structures.

The related MSM models stand up to various statistical tests, and the state-to-state dynamics they reveal for the free and antigen-bound antibody allow for drawing interesting insights. The MSMs show that, with antigen binding, there are considerable state-to-state transitions. These suggest that the antigen-bounded form may provide many dynamic processes to further enhance co-factor binding of the antibody in the next step. We also observe that the antigen binding reduces the number of macro-states. Actually, these insights suggest that the directions we investigated in this paper advance our understanding of the relationship between antibody dynamics and antibody–antigen recognition.

## Figures and Tables

**Table 1:** Run-time Comparison.

ID	Setting-A	Setting-B	Setting-C
Free Antibody	1 h 41 m	1 h 17 m	**26 m**
Antigen-bound Antibody	2 h 5 m	1 h 44 m	**29 m**

## Data Availability

All data and code that support the findings in this study are available to researchers upon reasonable request.

## Supplementary Materials

The following are available online at https://www.mdpi.com/article/10.3390/biom12071011/s1, Figures S1–S12 demonstrate the deviation in terms of backbone RMSD of the entire set of structures (160,000 structures) with respect to each of the 12 starting/seed structures. Figures S13–S15 relate the results obtained from conducting the CK test over the MSM models obtained, respectively, from Settings A–C for free antibody. Figures S16–S18 relate the results obtained from conducting the CK test over the MSM models obtained respectively from Settings A–C for the antigen-bound antibody.

## Abbreviations

The following abbreviations are used in this manuscript:

CA Atom	Carbon Alpha Atom
CK Test	Chapman–Kolmogorov Test
GBMV	Generalized Born method using Molecular Volume
MD	Molecular Dynamics
MSM	Markov State Model
PCA	Principal Component Analysis
TICA	Time-lagged Independent Component Analysis
USR	Ultrafast Shape Recognition
