# Supplementary material for: Fewer Dimensions, More Structures for Improved Discrete Models of Dynamics of Free versus Antigen-Bound Antibody
Source: Biomolecules. 2022 Jul 21;12(7):1011. doi: 10.3390/biom12071011 (PMC9313177; doi:10.3390/biom12071011)
Supplement: Supplementary file 1 [file biomolecules-12-01011-s001.zip › biomolecules-1774792-supplementary.pdf]

Fewer Dimensions, More Structures for Improved Discrete Models of Dynamics of Free Versus Antigen-Bound Antibody

Kazi Lutful Kabir, Buyong Ma, Ruth Nussinov, and Amarda Shehu

Deviation of Structures from Initial/Starting Structures for the Free Antibody System

We demonstrate how far the $160,000$ MD-generated structures (collected over all 12 MD trajectories) deviate from each of the 12 initial/seed structures. For each generated structure, we calculate its backbone RMSDs from a given starting structure, and show the backbone RMSDs in the plots below. We also record the maximum backbone RMSD achieved in each plot.

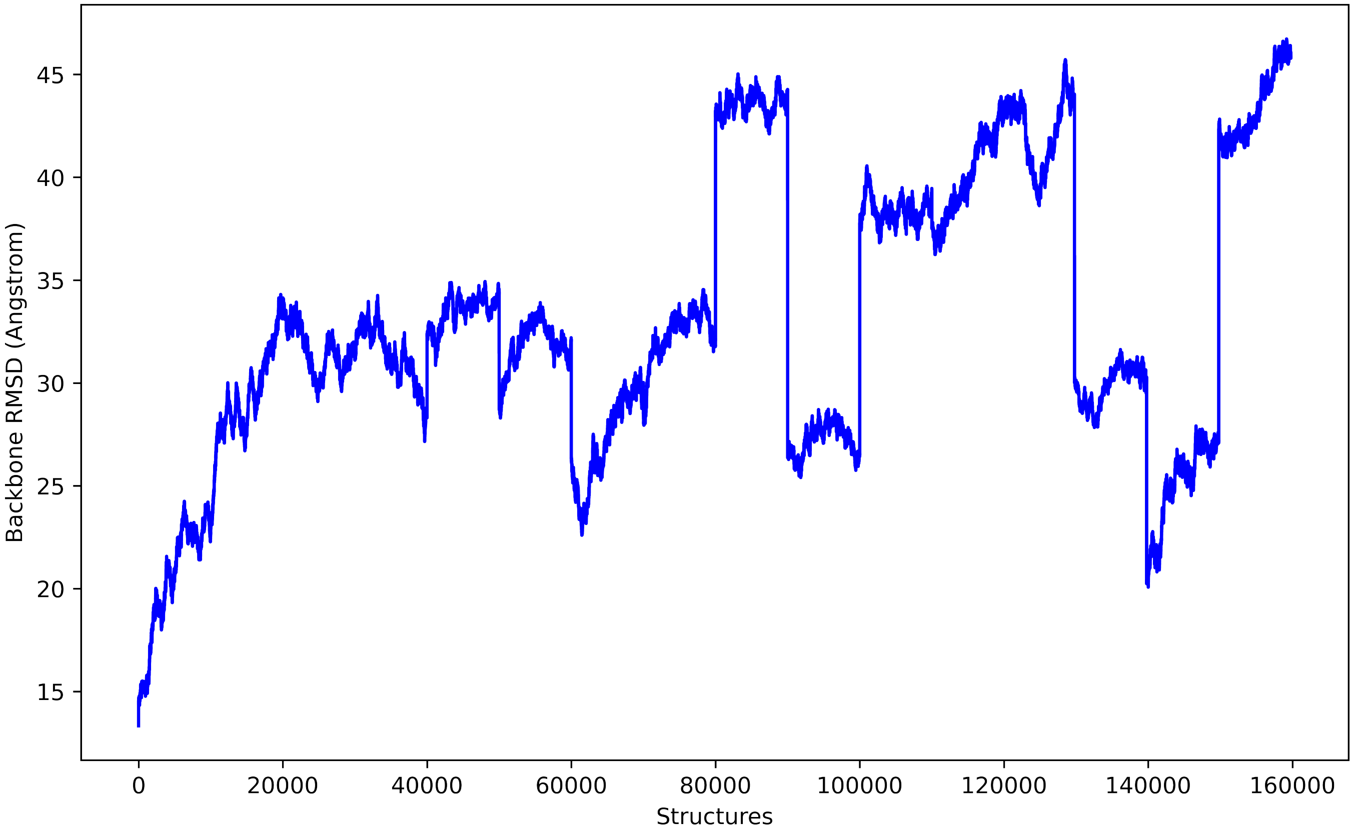

Figure S1: We draw the backbone RMSDs of the $160,000$ MD-generated structures from the structure seeding MD trajectory 1/12. The maximum RMSD of 46.725Å is recorded at the $159,191^{th}$ frame.

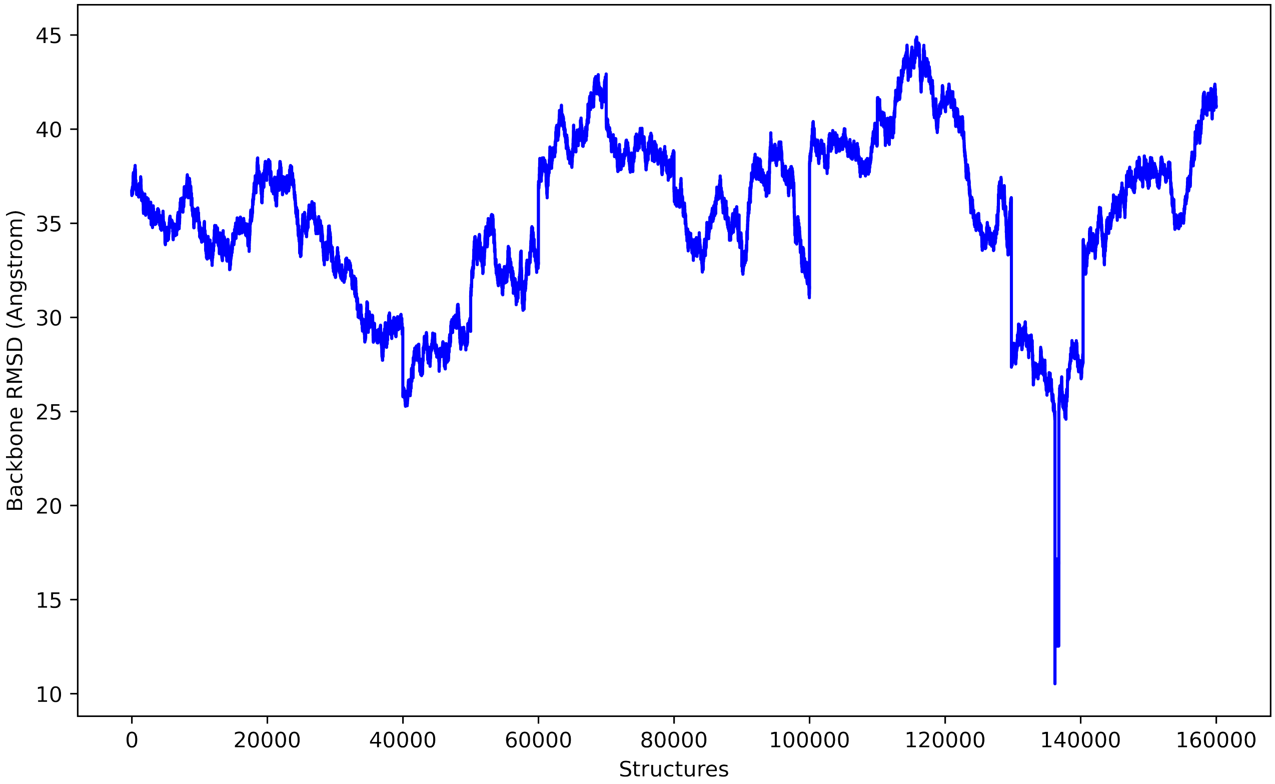

Figure S2: We draw the backbone RMSDs of the 160,000 MD-generated structures from the structure seeding MD trajectory 2/12. The maximum RMSD of 44.886Å is recorded at the 115,808th frame.

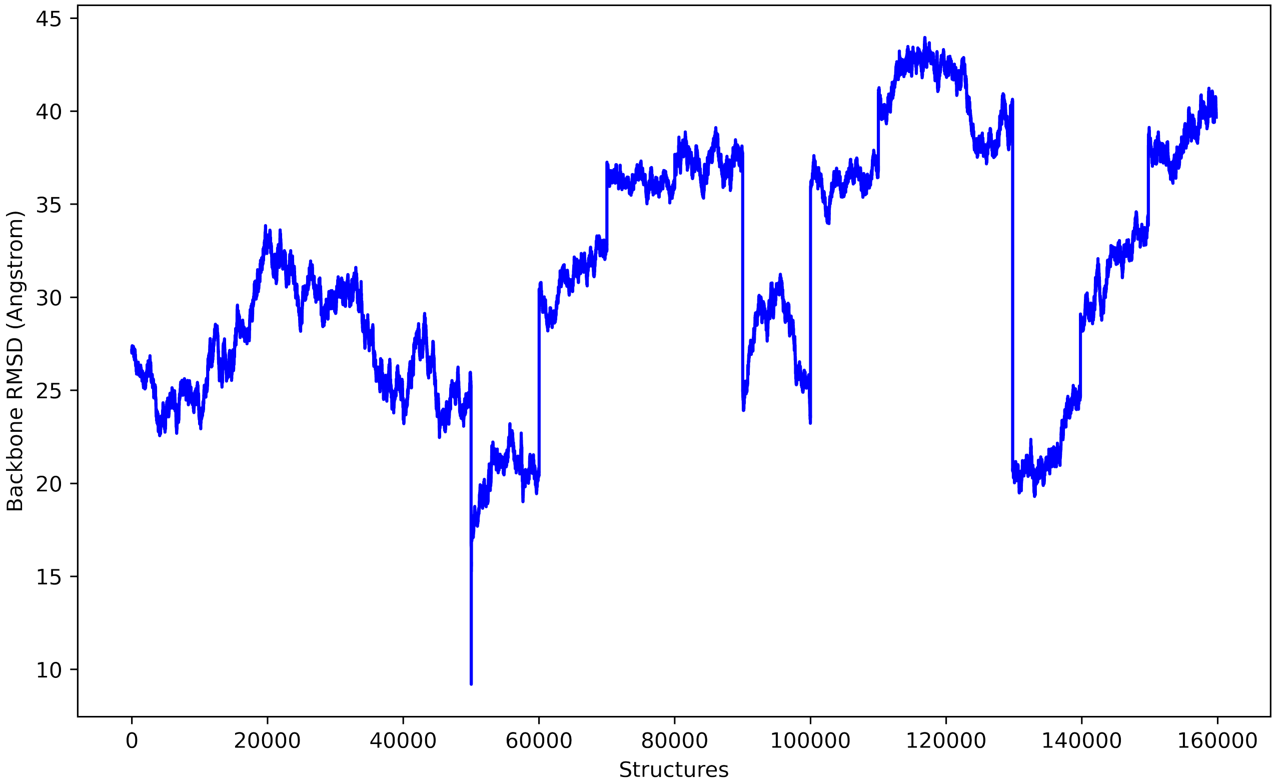

Figure S3: We draw the backbone RMSDs of the 160,000 MD-generated structures from the structure seeding MD trajectory 3/12. The maximum RMSD of 43.954Å is recorded at the 116,861th frame.

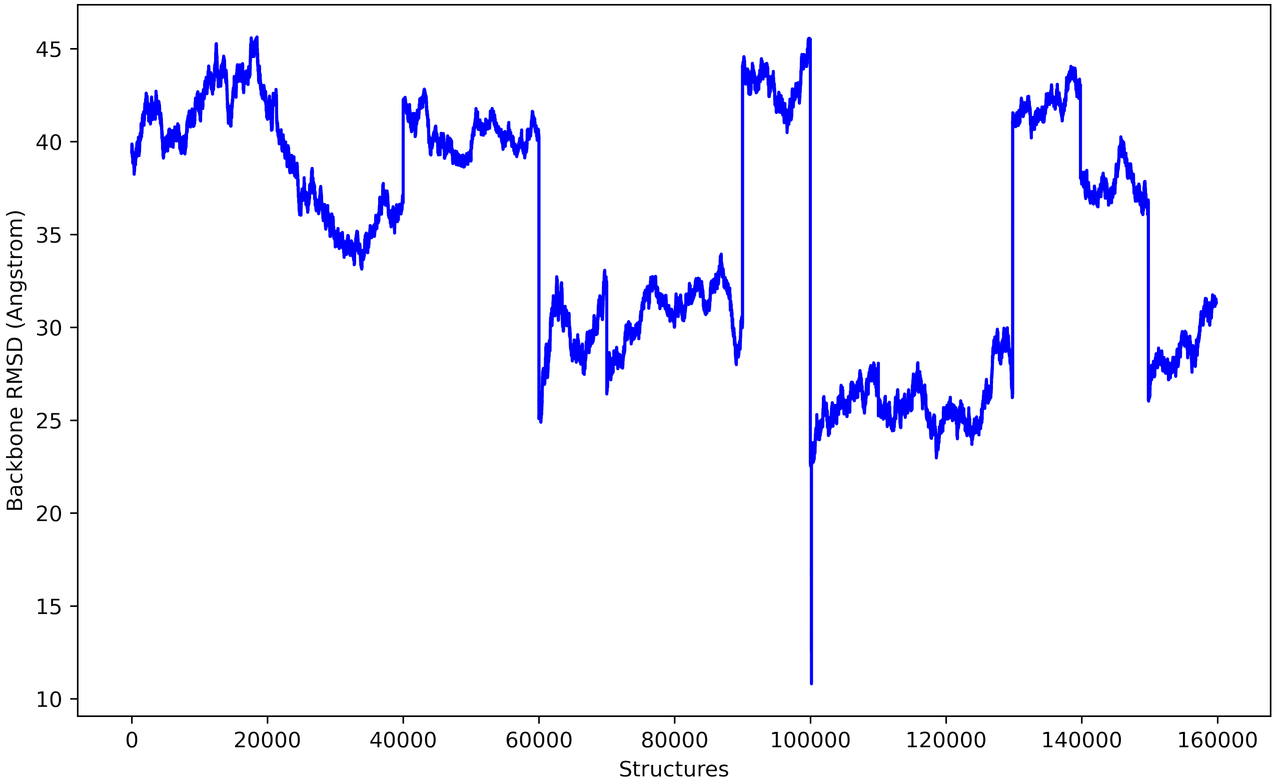

Figure S4: We draw the backbone RMSDs of the 160,000 MD-generated structures from the structure seeding MD trajectory 4/12. The maximum RMSD of 45.633Å is recorded at the 18,453th frame.

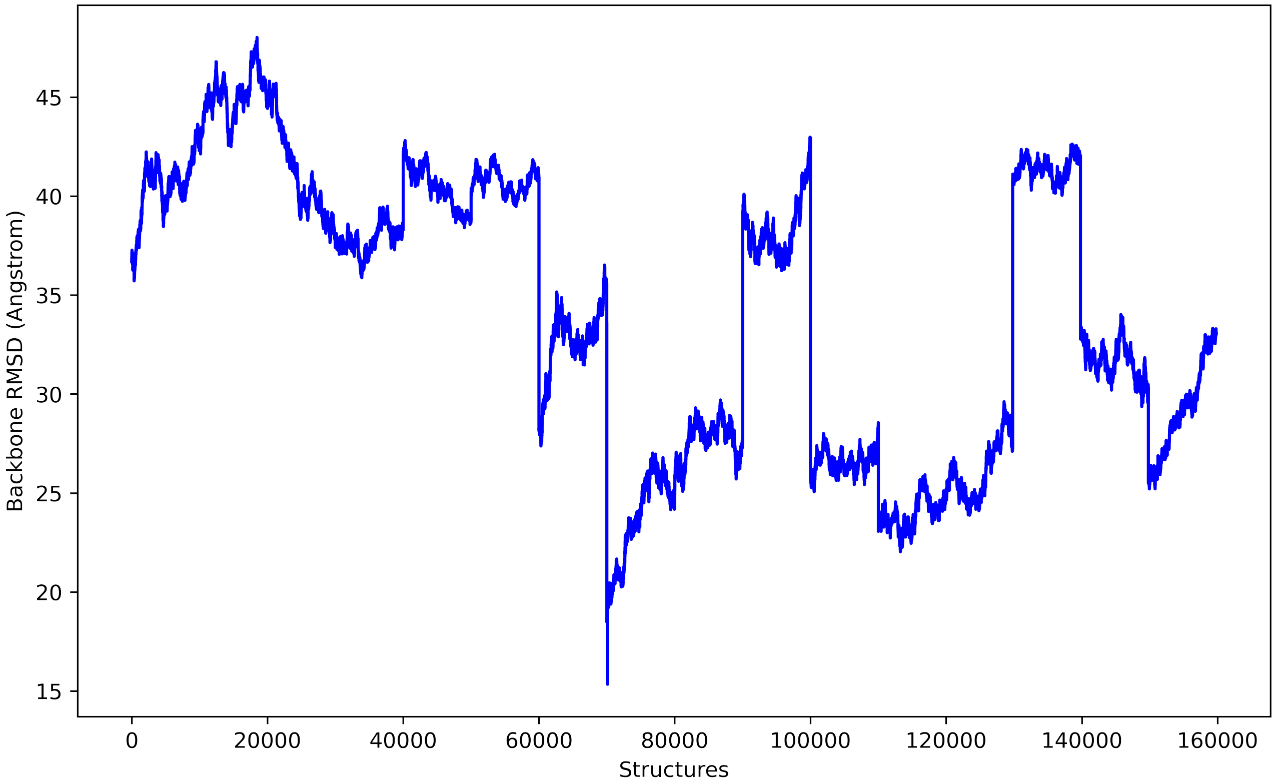

Figure S5: We draw the backbone RMSDs of the 160,000 MD-generated structures from the structure seeding MD trajectory 5/12. The maximum RMSD of 48.016Å is recorded at the 18,453th frame.

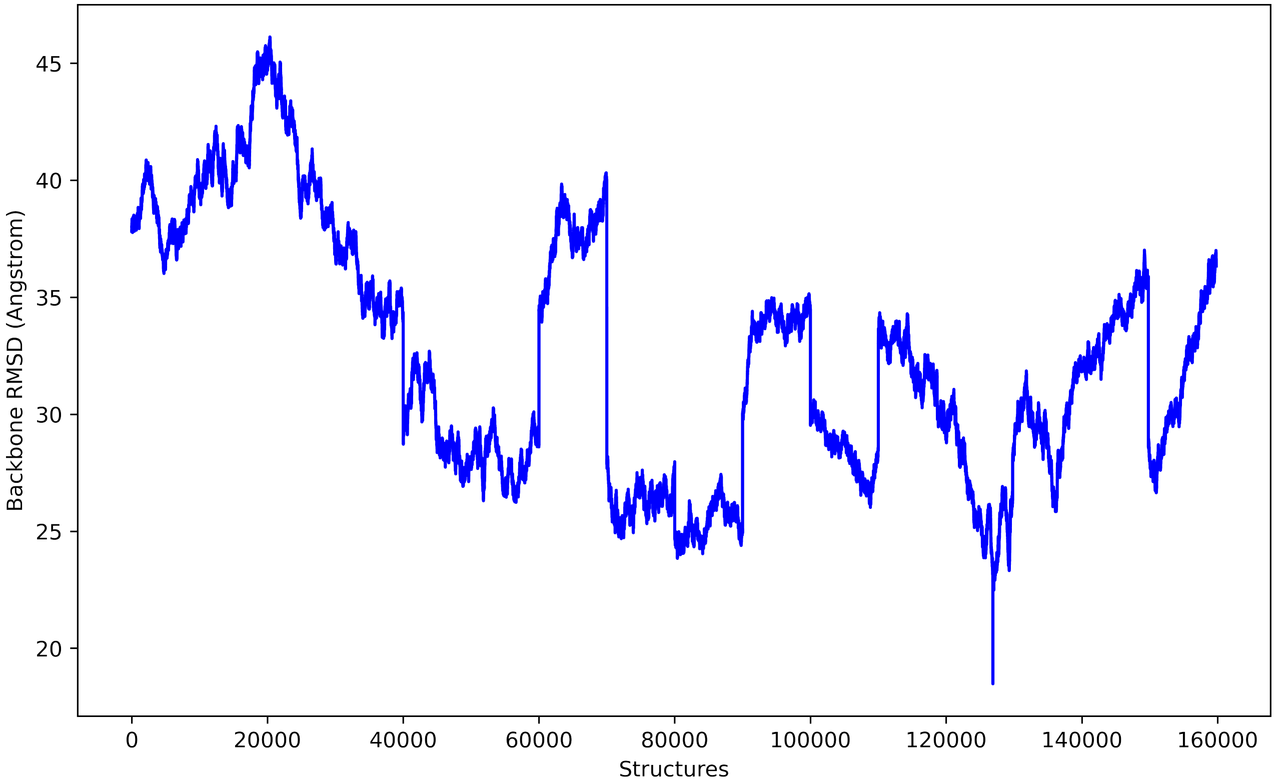

Figure S6: We draw the backbone RMSDs of the 160, 000 MD-generated structures from the structure seeding MD trajectory 6/12. The maximum RMSD of 46.124Å is recorded at the 20,357th frame.

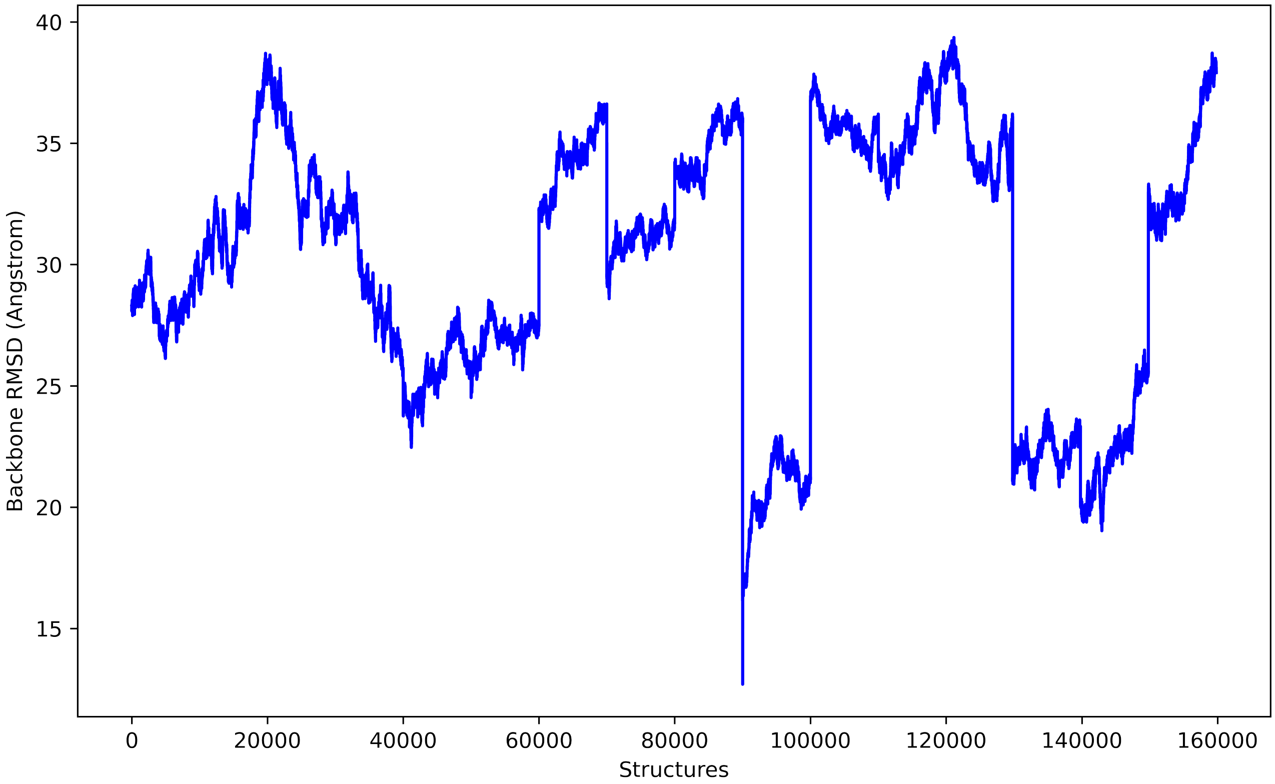

Figure S7: We draw the backbone RMSDs of the 160, 000 MD-generated structures from the structure seeding MD trajectory 7/12. The maximum RMSD of 39.357Å is recorded at the 12,1145th frame.

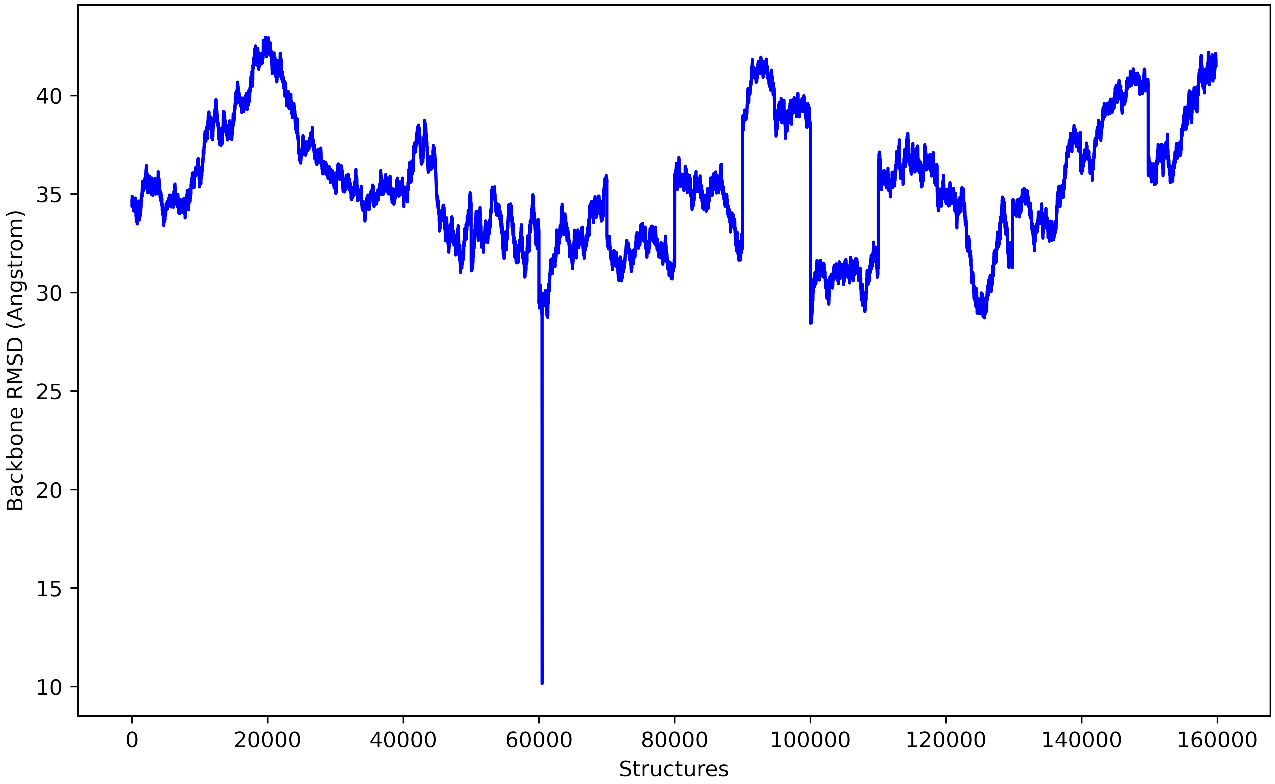

Figure S8: We draw the backbone RMSDs of the $160,000$ MD-generated structures from the structure seeding MD trajectory 8/12. The maximum RMSD of 42.962Å is recorded at the 19,711th frame.

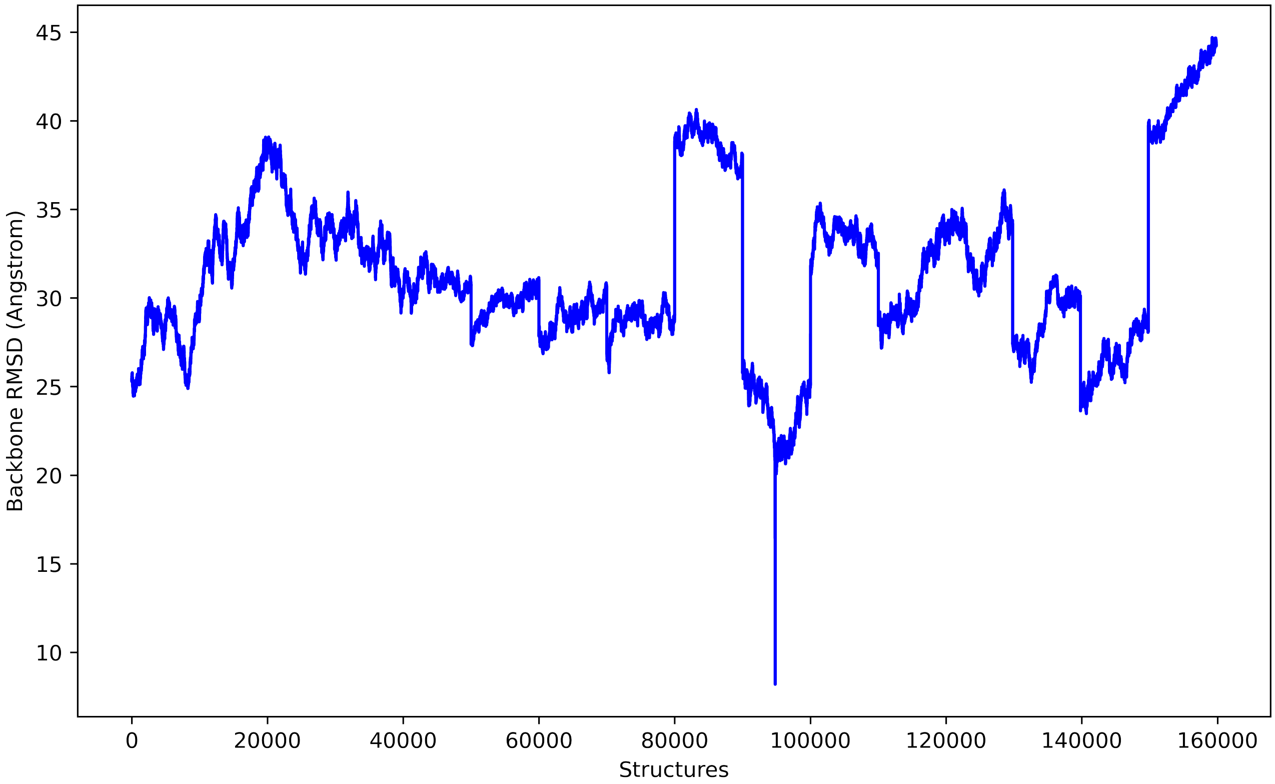

Figure S9: We draw the backbone RMSDs of the $160,000$ MD-generated structures from the structure seeding MD trajectory 9/12. The maximum RMSD of 44.701Å is recorded at the 159,207th frame.

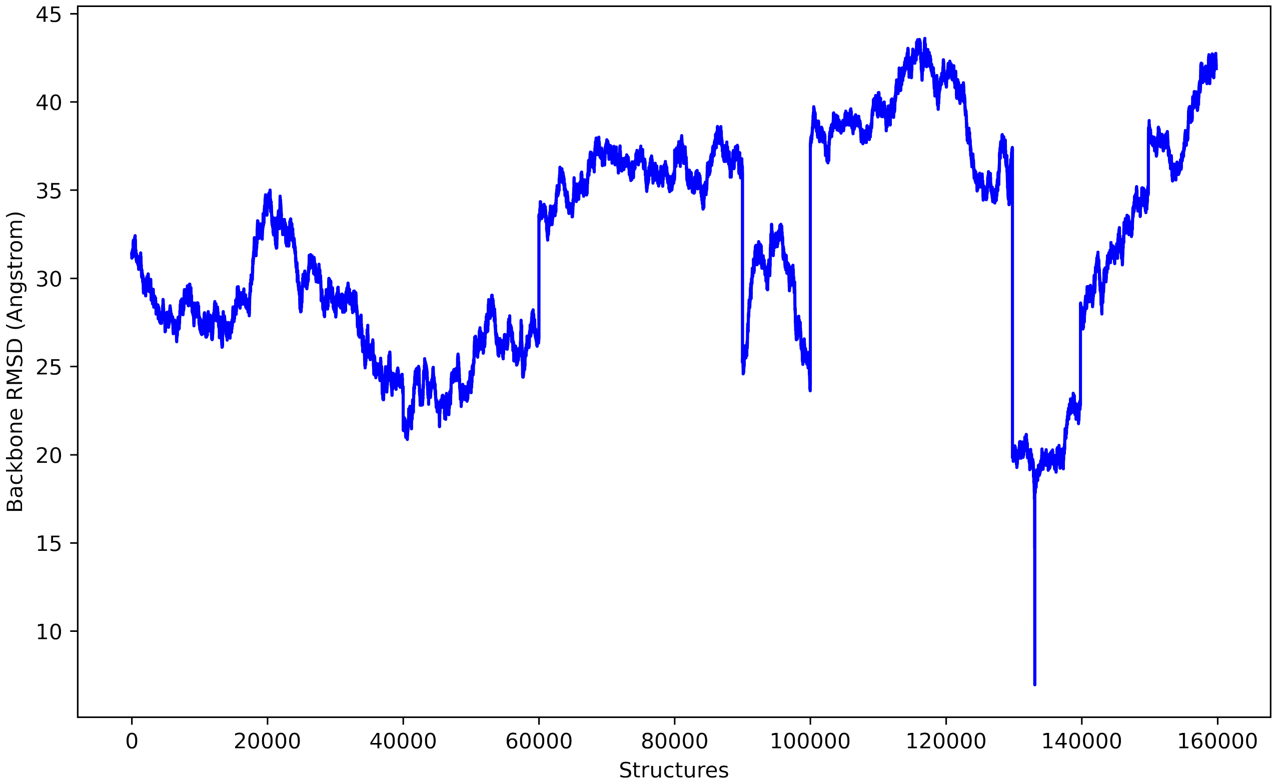

Figure S10: We draw the backbone RMSDs of the 160, 000 MD-generated structures from the structure seeding MD trajectory 10/12. The maximum RMSD of 43.603Å is recorded at the 116,871th frame.

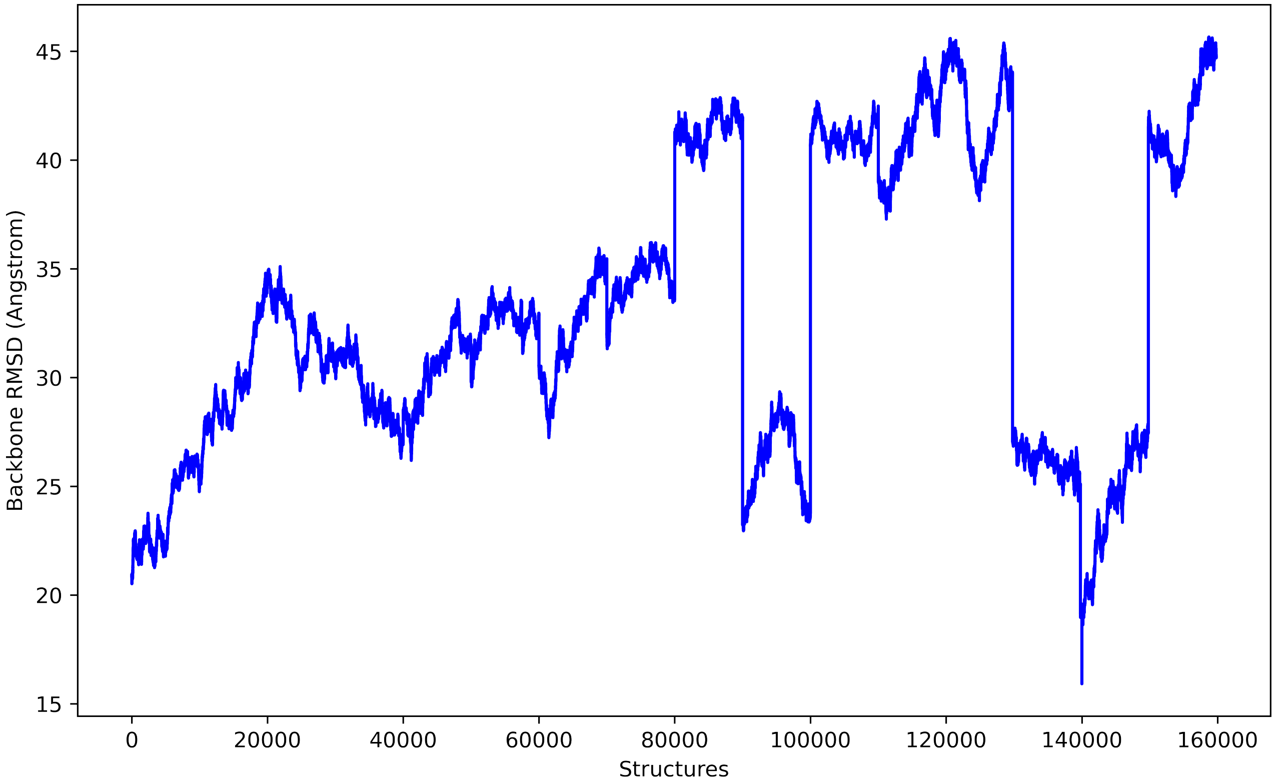

Figure S11: We draw the backbone RMSDs of the $160,000$ MD-generated structures from the structure seeding MD trajectory 11/12. The maximum RMSD of 45.66Å is recorded at the 158,710th frame.

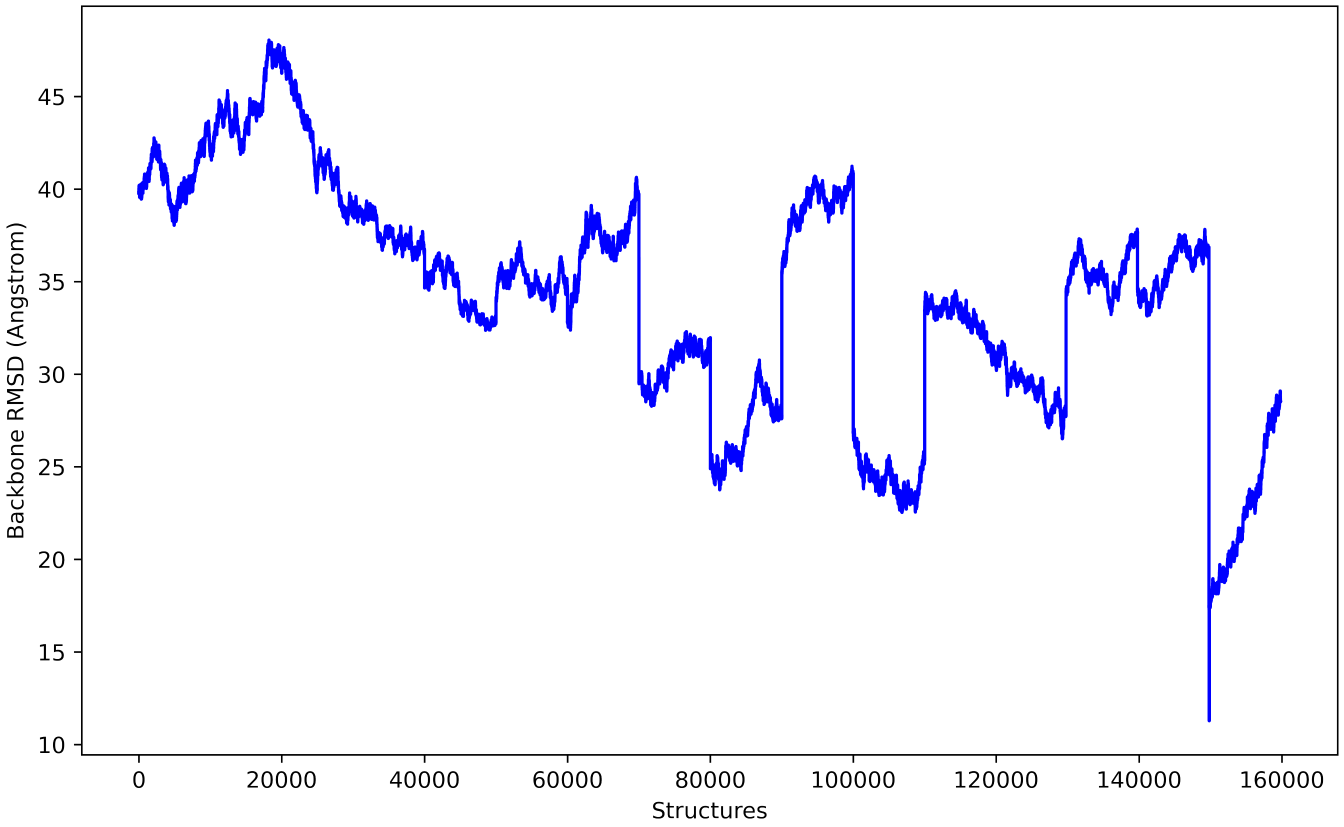

Figure S12: We draw the backbone RMSDs of the 160, 000 MD-generated structures from the structure seeding MD trajectory 12/12. The maximum RMSD of 48.04Å is recorded at the 18,222th frame.

MSM Evaluation via the CK Test

Free Antibody MSM Evaluation via the CK Test

Fig. S13- S15 relate the results obtained from conducting the CK test over the MSM models obtained respectively from Settings A-C for the free antibody. Specifically, these figures visualize the outcome for the five states identified in each setting that have the highest self-transition probabilities. The plots show deviations between the estimated and the predicted model in Settings A-C. These are largest under Setting-A and gradually reduce over Setting-B and Setting-C; the deviations are negligible for Setting-C. In good agreement with the convergence analysis test related in the main manuscript, the CK test additionally confirms that the MSM model obtained from Setting-C is the best, as it stands best to both of these statistical tests.

Antigen-bound Antibody MSM Evaluation via the CK Test

Fig. S16-S18 relate the results obtained from conducting the CK test over the MSM models obtained respectively from Settings A-C for the antigen-bound antibody. As for the antigen-bound antibody, the figures visualize the outcome for the five states identified in each setting that have the highest self-transition probabilities. The plots show deviations between the estimated and the predicted model in Settings A-C. They show that these are largest under Setting-A and gradually reduce over Setting-B and Setting-C. Again, the deviations are negligible for Setting-C, as for the free antibody. In good agreement with the convergence analysis test related above, the CK test additionally confirms that the MSM model obtained for the antigen-bound antibody from Setting-C stands best to both of the statistical tests (convergence analysis and CK-test).

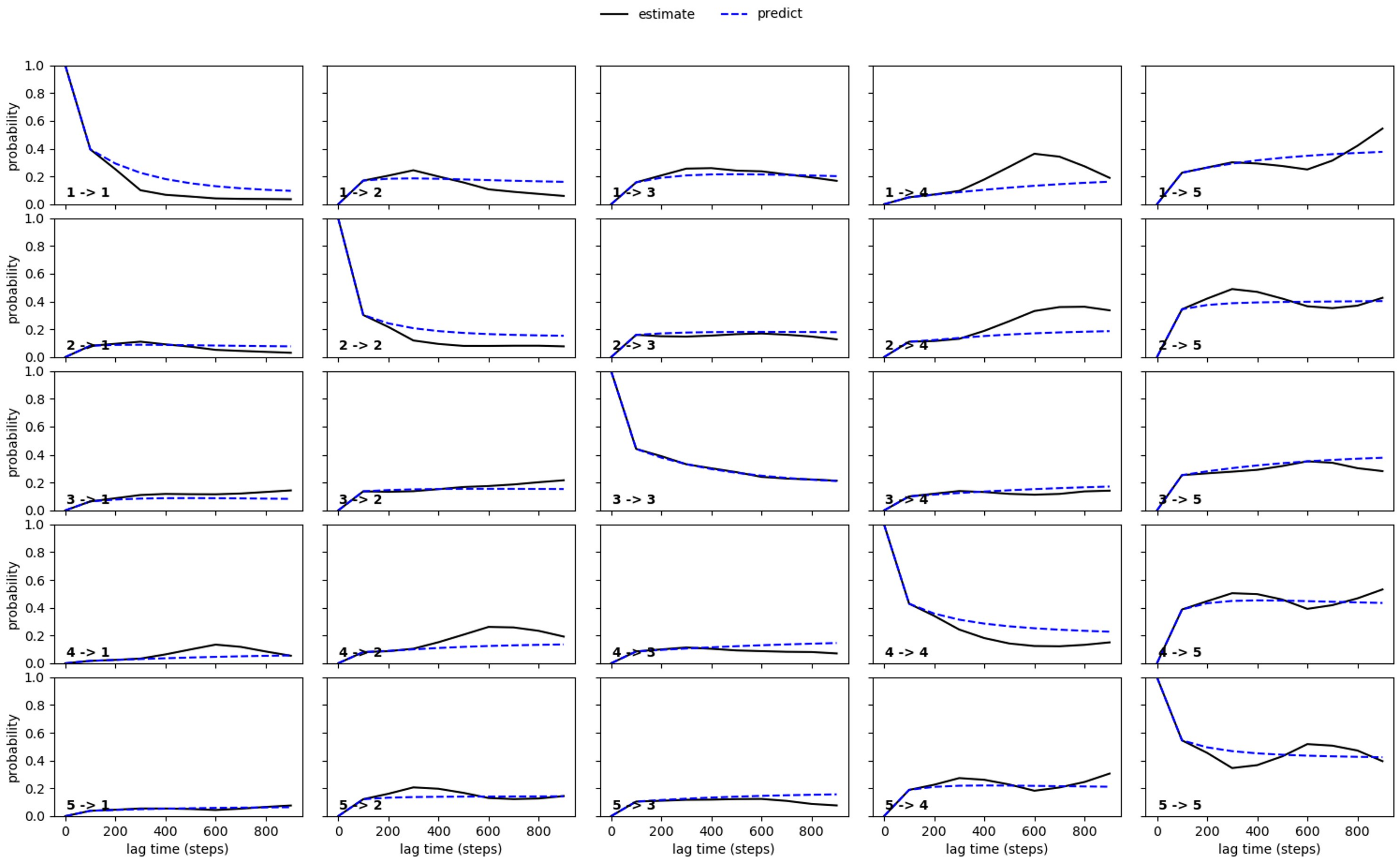

Figure S13: The CK test is carried out on the free antibody MSM model obtained from Setting-A. Deviations between the estimated and the predicted model are large in Setting-A

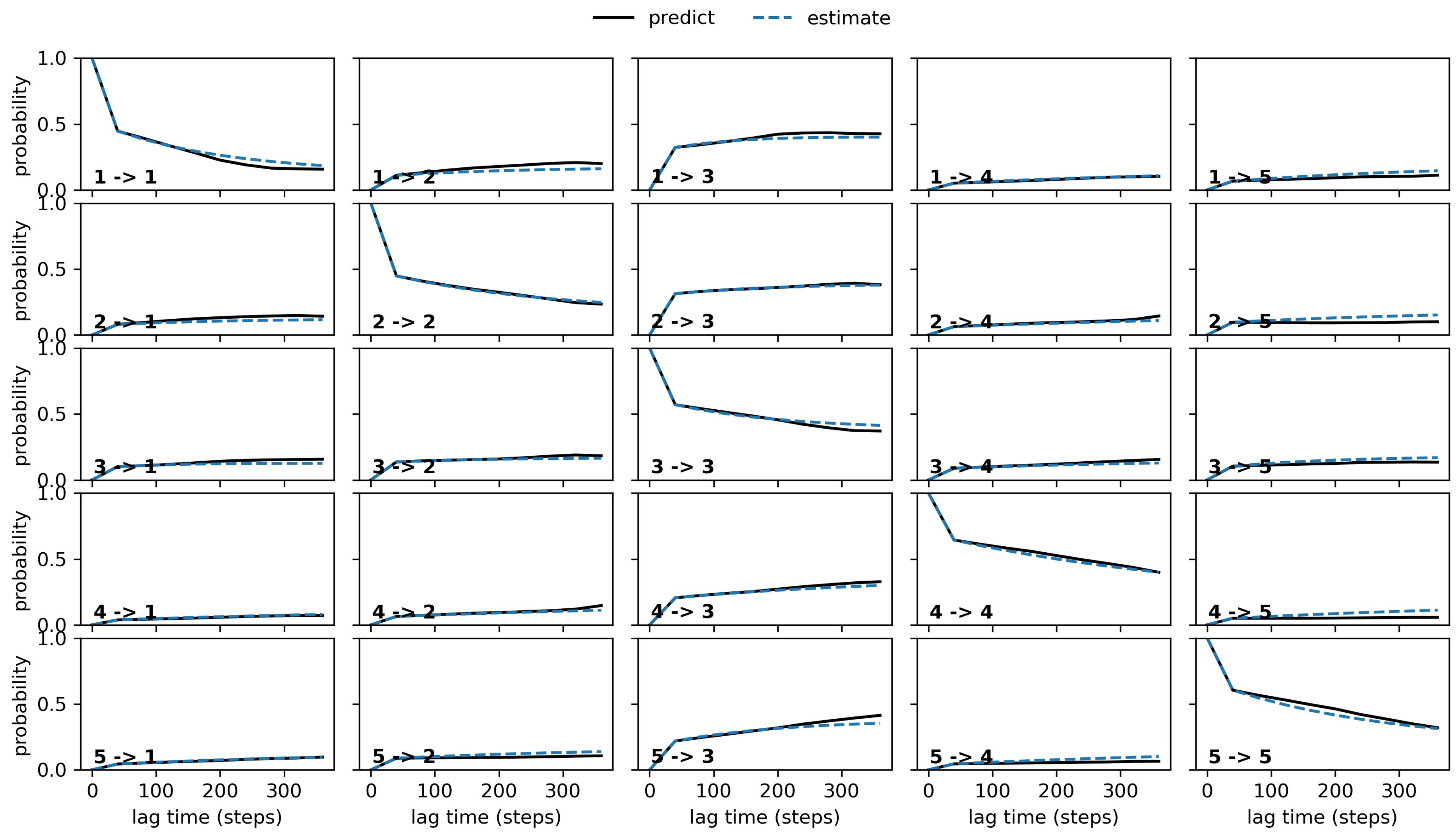

Figure S14: The CK test is carried out on the free antibody MSM model obtained from Setting-B. Deviations between the estimated and the predicted model are less than setting-A

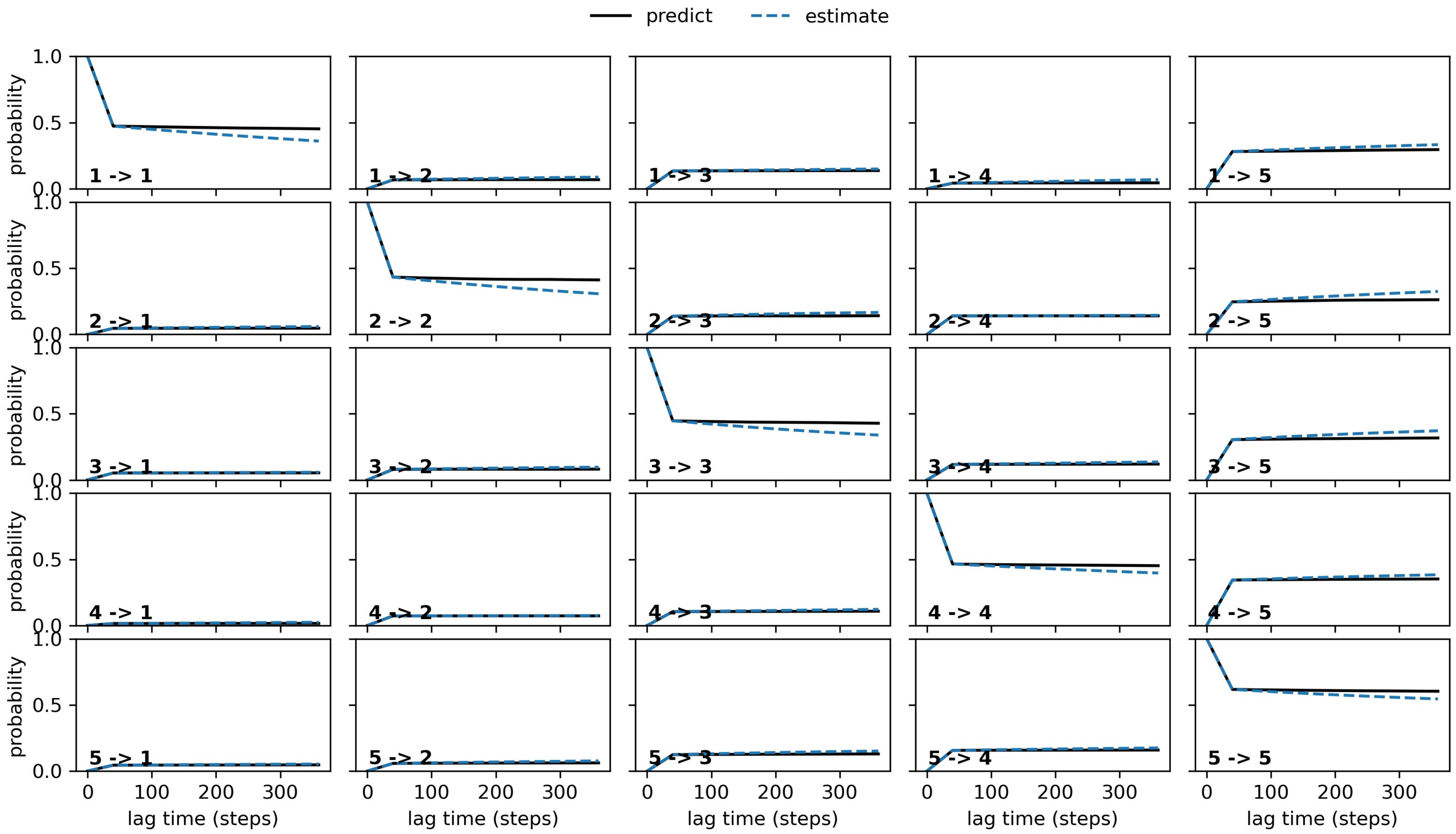

Figure S15: The CK test is carried out on the free antibody MSM model obtained from Setting-C. Deviations between the estimated and the predicted model are negligible

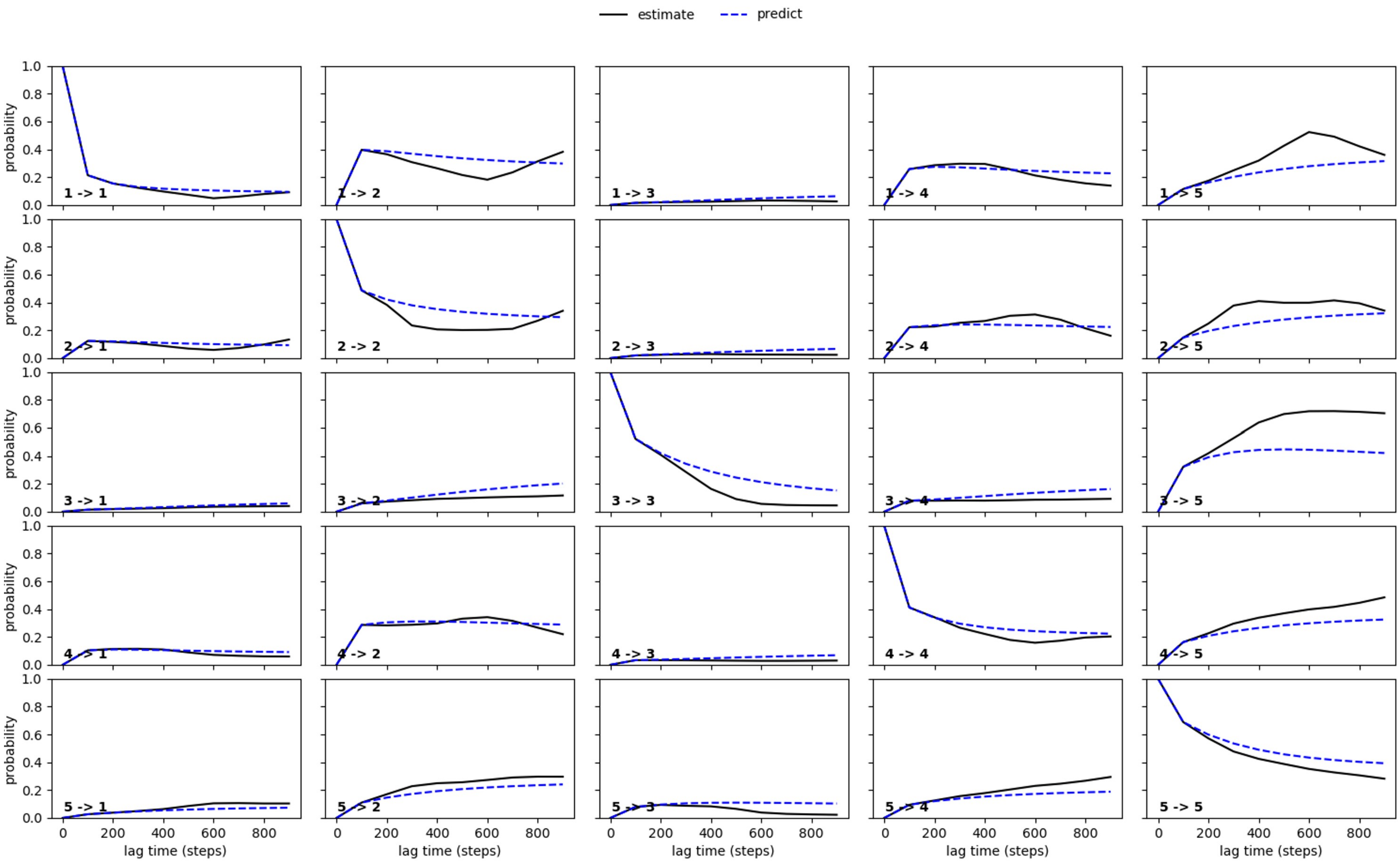

Figure S16: The CK test is carried out on the antigen-antibody complex MSM model obtained from Setting-A. Deviations between the estimated and the predicted model are large in Setting-A

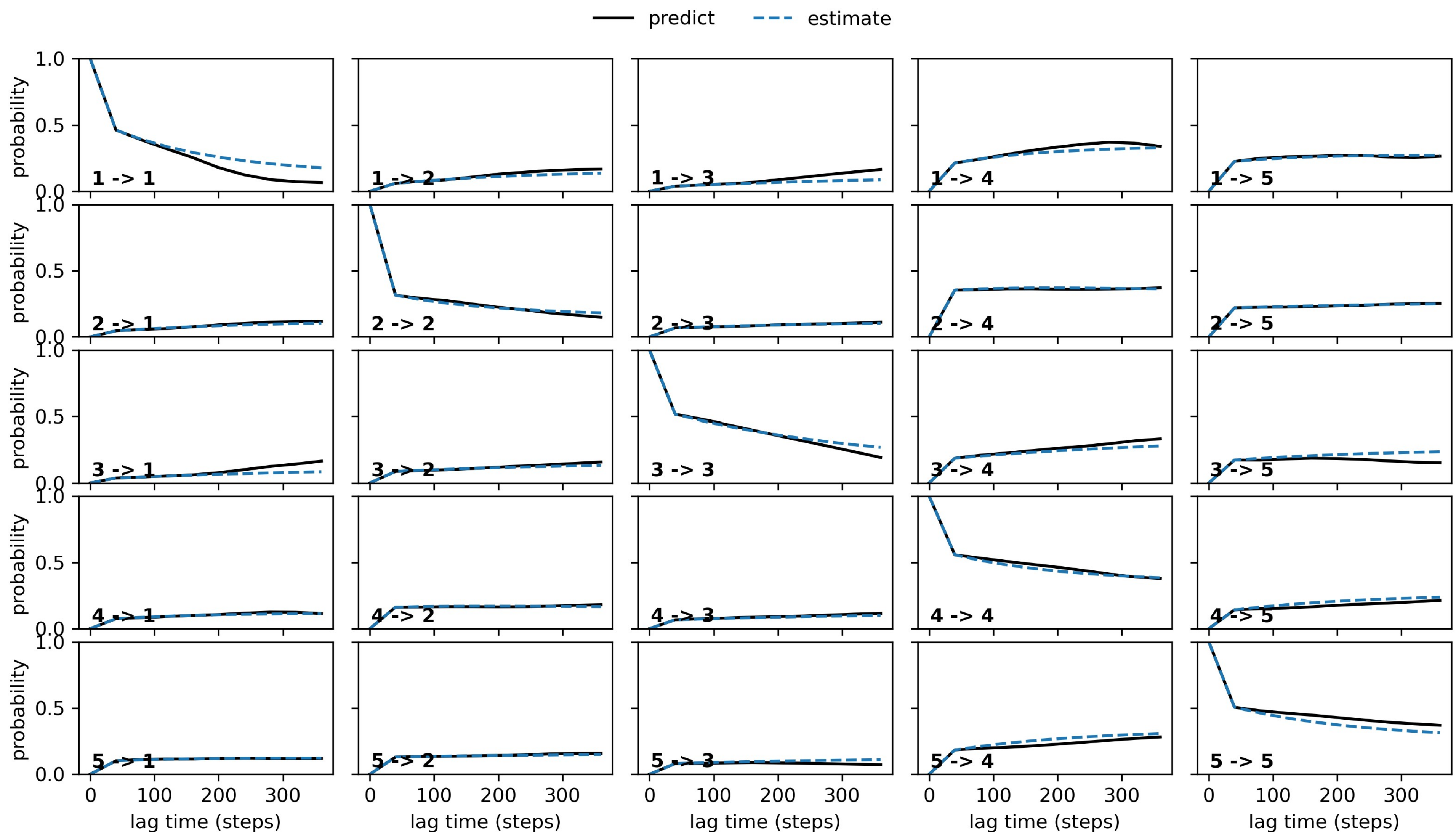

Figure S17: The CK test is carried out on the antigen-antibody complex MSM model obtained from Setting-B. Deviations between the estimated and the predicted model are less than setting-A

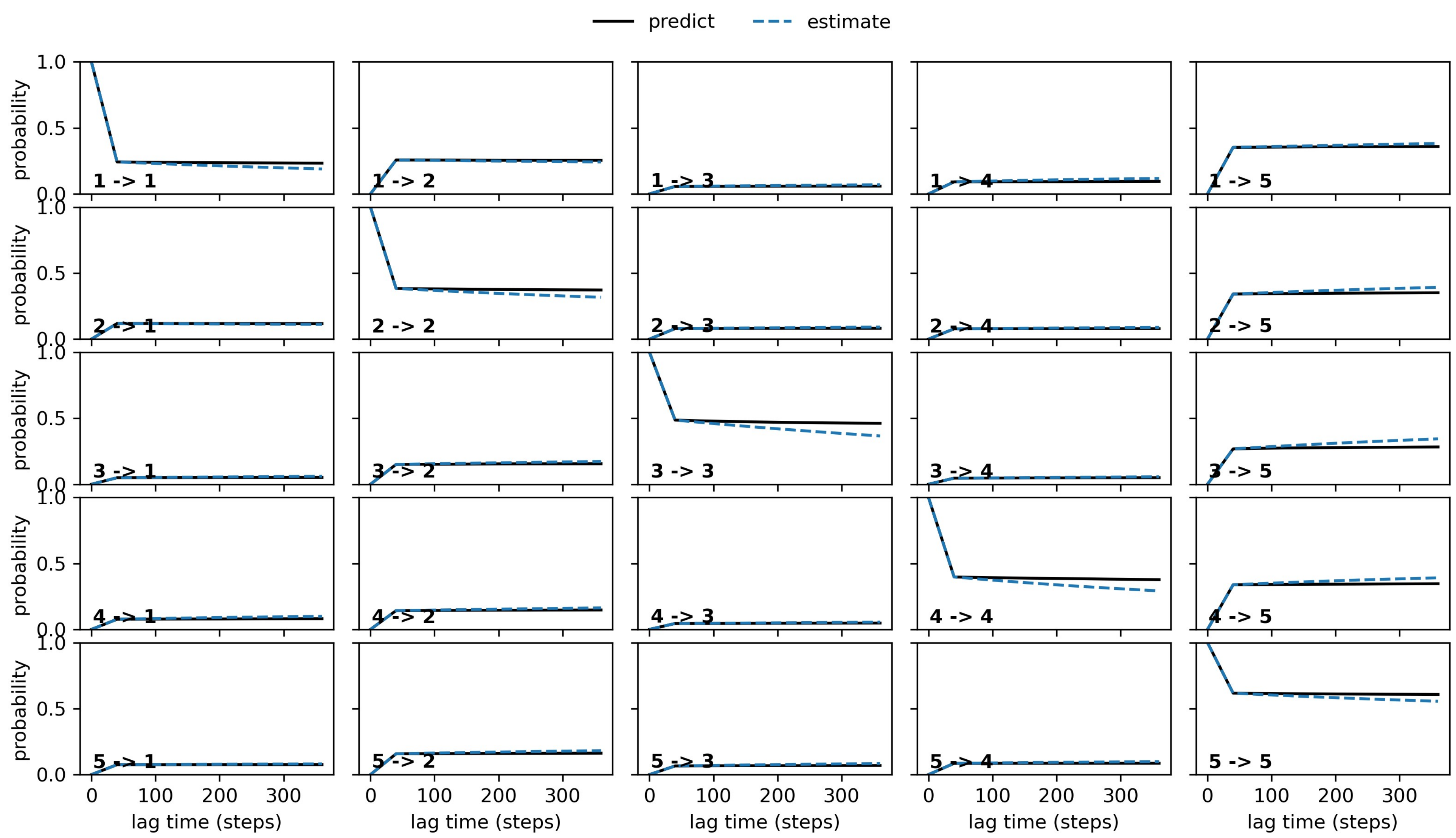

Figure S18: The CK test is carried out on the antigen-antibody complex MSM model obtained from Setting-C. Deviations between the estimated and the predicted model are negligible
